# Evolutionary Spread of Distinct *O*‐methyltransferases Guides the Discovery of Unique Isoaspartate‐Containing Peptides, Pamtides

**DOI:** 10.1002/advs.202305946

**Published:** 2023-11-20

**Authors:** Hyunbin Lee, Sho Hee Park, Jiyoon Kim, Jaehak Lee, Min Sun Koh, Jung Ho Lee, Seokhee Kim

**Affiliations:** ^1^ Department of Chemistry Seoul National University 1 Gwanak‐ro, Gwanak‐gu Seoul 08826 Republic of Korea

**Keywords:** genome mining, graspetides, pamtides, protein L‐(iso)aspartyl O‐methyltransferase, RiPP

## Abstract

Ribosomally synthesized and post‐translationally modified peptides (RiPPs) are a structurally diverse class of natural products with a distinct biosynthetic logic, the enzymatic modification of genetically encoded precursor peptides. Although their structural and biosynthetic diversity remains largely underexplored, the identification of novel subclasses with unique structural motifs and biosynthetic pathways is challenging. Here, it is reported that peptide/protein L‐aspartyl *O*‐methyltransferases (PAMTs) present in several RiPP subclasses are highly homologous. Importantly, it is discovered that the apparent evolutionary transmission of the PAMT gene to unrelated RiPP subclasses can serve as a basis to identify a novel RiPP subclass. Biochemical and structural analyses suggest that homologous PAMTs convert aspartate to isoaspartate via aspartyl‐*O*‐methyl ester and aspartimide intermediates, and often require cyclic or hairpin‐like structures for modification. By conducting homology‐based bioinformatic analysis of PAMTs, over 2,800 biosynthetic gene clusters (BGCs) are identified for known RiPP subclasses in which PAMTs install a secondary modification, and over 1,500 BGCs where PAMTs function as a primary modification enzyme, thereby defining a new RiPP subclass, named pamtides. The results suggest that the genome mining of proteins with secondary biosynthetic roles can be an effective strategy for discovering novel biosynthetic pathways of RiPPs through the principle of “guilt by association”.

## Graphical abstract

Comprehensive bioinformatic analysis demonstrates the close evolutionary relationship of peptide/protein L‐aspartyl *O*‐methyltransferases (PAMTs) found in biosynthetic gene clusters for ribosomally synthesized and post‐translationally modified peptides (RiPPs). Genome mining, heterologous expression, mass analysis, and multidimensional nuclear magnetic resonance analyses uncover pamtides, a unique subclass of RiPPs containing isoaspartyl moiety.

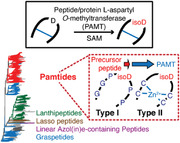

## 1 Introduction

Ribosomally synthesized and post‐translationally modified peptides (RiPPs) are a rapidly expanding class of natural products.^[^ ^1^ ^]^ RiPPs exhibit a unique biosynthetic pathway that involves genetically encoded precursor peptides and enzymes, with the latter installing diverse post‐translational modifications (PTMs) on the precursor peptides. The precursor peptides typically have two modules: the leader and core regions, which are recognized and modified by PTM enzymes, respectively. This biosynthetic simplicity and modularity, and thereby high evolvability, underlies not only the structural and functional diversity of RiPPs but also the high potential for engineering novel functional biomolecules.

The hallmark of RiPP diversity is the distinct PTMs and associated biosynthetic pathways, which individually define the subclasses of RiPPs. The number of RiPP subclasses has increased from 23 in 2013^[^ ^1a^ ^]^ to 41 in 2021,^[^ ^1b^ ^]^ highlighting the recent expansion of the RiPP diversity, which is likely to continue in the near future. Discovery of new RiPP subclasses has traditionally relied on fortuitous isolation by activity‐based screening.^[^ ^1a^ ^]^ While the explosion of genome sequence data and development of genome mining tools have allowed the identification of a large number of putative biosynthetic gene clusters (BGCs) for RiPPs,^[^ ^2^ ^]^ these methods are generally inefficient at uncovering novel RiPP subclasses; unlike nonribosomal peptide synthetases (NRPSs), RiPP biosynthetic enzymes lack common features across all RiPP subclasses, and therefore, the typical genome mining approach based on homology of biosynthetic enzymes is mostly limited to the expansion of known or closely related RiPP subclasses.

Dissecting phylogenetically distinct orphan BGCs has proven effective for revealing novel PTMs and RiPP‐associated pathways, exemplified by recent discoveries of spliceotides,^[^ ^3^ ^]^ ranthipeptides,^[^ ^4^ ^]^ streptides,^[^ ^5^ ^]^ pearlins,^[^ ^6^ ^]^ and triceptides.^[^ ^7^ ^]^ However, this approach depends on the functional divergence of enzymes known for other class‐defining PTMs and has particularly been successful with an enzyme family with versatile activities, radical *S*‐adenosylmethionine (rSAM) enzymes.^[^ ^8^ ^]^ Furthermore, it is often challenging to find candidate BGCs that significantly diverge from those responsible for known PTMs. To bypass the requirement for homologous biosynthetic enzymes, a marker‐independent strategy called decRiPPter has recently emerged.^[^ ^9^ ^]^ This method uncovered a new subfamily of lanthipeptides, but it may be biased on the characteristics of known RiPP precursors and requires further exploration to unveil novel PTMs. Overall, new strategies are needed to systematically uncover the unexplored chemical and biosynthetic space associated with RiPPs.

**Figure 1:**
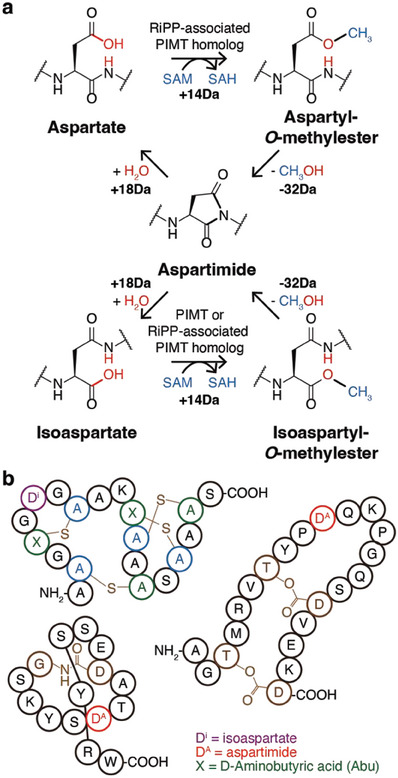
Overview of PIMT homolog‐associated peptide maturation. a) Molecular mechanism of reactions by PIMTs and homologs. Isoaspartates (bottom left) are methylated by PIMTs or their homologs to yield isoaspartyl‐*O*‐methylesters (bottom right). Aspartates are similarly transformed to aspartyl‐*O*‐methylesters by the homologs. Nitrogen in the backbone amide of the methylesters attacks the carbonyl group to yield aspartimides (middle), and these can be hydrolyzed either to aspartates or isoaspartates. Accompanying molecular weight changes in these conversions are written below; atoms that added or removed in each step are colored in blue (methyl group from SAM) or red (water molecule or hydroxyl group in methanol). b) Structure of PIMT homolog‐modified lanthipeptide (OlvA(BCS_A_)^GluC^, top left), lasso peptide (lihuanodin, bottom left), and graspetide (fuscimiditide, right). Isoaspartate and aspartimides are highlighted in purple and red, respectively. D‐Aminobutyric acids in the lanthipeptide are denoted as “X” and colored in green. Class‐defining modifications are colored in brown.

Here, using homologs of protein L‐(iso)aspartyl *O*‐methyltransferases (PIMTs), we demonstrate that the genome mining of a secondary modification enzyme can promote the identification of a novel RiPP subclass. PIMTs, also known as L‐isoaspartyl protein carboxyl methyltransferases (PCMs), were originally discovered as repair enzymes for damaged proteins. They catalyze the conversion of isoaspartate (isoAsp), which is spontaneously formed from an Asp or Asn residue, to Asp (**Figure** **1**a).^[^ ^10^ ^]^ Recently, PIMT homologs were found in BGCs of several RiPP subclasses, including lanthipeptides, lasso peptides, and graspetides, where they install secondary modifications on conserved Asp residues.^[^ ^11^ ^]^ This results in the formation of an aspartimide or isoAsp, the former of which can be spontaneously hydrolyzed to the latter or Asp (Figure 1).

We have expanded the structural diversity of the graspetide family of RiPPs, also known as omega‐ester‐containing peptides (OEPs).^[^ ^12^ ^]^ Graspetides and their biosynthesis have been also studied by bioinformatic,^[^ ^13^ ^]^ biochemical,^[^ ^14^ ^]^ and structural analyses.^[^ ^15^ ^]^ Using heterologous co‐expression, in vitro reconstitution of enzyme reactions, and structural analyses of the product peptides with mass spectrometry and nulear magnetic resonance (NMR), we discovered that SsfM, a PIMT homolog associated with graspetide biosynthesis, has the same activity as other PIMT homologs in RiPP biosynthesis. Genome mining of PIMT homologs revealed not only homologous enzymes playing a secondary role for known RiPP subclasses but also those mediating the same conversion of Asp as the primary modification, leading to a new RiPP subclass. This result highlights the potential of leveraging the evolutionary dissemination of tailoring enzymes across diverse RiPP BGCs to identify a novel biosynthetic pathway of RiPPs.

## 2 Results and Discussion

### 2.1 *O*‐methyltransferase‐Associated Gene Cluster Produces an Isoaspartate‐Containing Pentacyclic Graspetide

**Figure 2:**
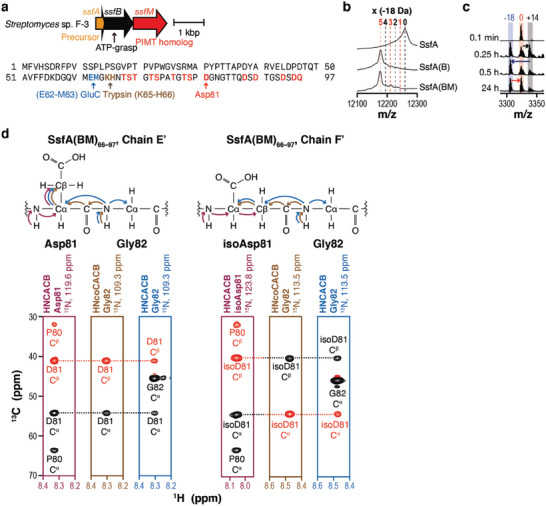
A PIMT homolog in the model BGC generates isoaspartate in a graspetide. a) Selected model BGC (top) and precursor peptide sequence (bottom). Arrows are color‐coded based on the predicted domains in the proteins. Cleavage sites by either GluC or trypsin are designated by arrows under the precursor sequence. Residues with hydroxyl or acidic side chains are highlighted in red. b) MALDI‐TOF‐MS spectra of purified SsfA, SsfA(B), and SsfA(BM). c) MALDI‐TOF‐MS spectra of the SsfM‐mediated modification of SsfA(B)_63–97_ in vitro. Substrates (20 µm) were mixed with SsfM (5 µm) in the presence of SAM (1 mm), DTT (1 mm), and Tris‐HCl pH 8.0 (50 mm) at 25 °C. Reactions were quenched at designated time points and monitored by a mass analyzer. Relative mass values to SsfA(B)_63–97_ are indicated above peaks. d) Strip plots of HNCACB and HNcoCACB spectra showing the presence of isoaspartate in SsfA(BM). Magnetization transfer in Asp‐Gly and isoAsp‐Gly for HNCACB and HNcoCACB experiments are illustrated above. Positive and negative contours are colored in black and red, respectively. Chemical shift values, observed, and calculated mass values can be found in Supporting Dataset S1 (Supporting Information).

Previous genome mining studies of graspetides have revealed the high co‐occurrence of PIMT homologs in graspetide BGCs (group 13 graspetides).^[^ ^13^, ^16^ ^]^ Heterologous expression of genes in two BGCs yielded aspartimidylated graspetides.^[^ ^11c,d^ ^]^ Here, we tested another graspetide BGC from *Streptomyces* sp. F‐3, encoding a precursor peptide (SsfA), an ATP‐grasp enzyme (SsfB), and a PIMT homolog (SsfM; **Figure** **2**a). Heterologous co‐expression of SsfA and SsfB in *Escherichia coli* (*E. coli*) produced the SsfB‐modified SsfA, named SsfA(B), which was 90 Da lighter than SsfA (Figure 2b). Incubation of the GluC‐digested SsfA(B), SsfA(B)_63–97_, with methoxide resulted in up to five‐fold methanolysis, suggesting that SsfA(B)_63–97_ has five ester linkages (Figure S1a, Supporting Information). The tandem mass (MS/MS) analysis of the fivefold methanolized SsfA(B)_63–97_ suggested that the five ring‐forming carboxylates reside within the C‐terminal 12 residues, which have only four Asp residues and no Glu (Figure S1b, Supporting Information). This result suggests the involvement of the C‐terminal carboxylate in ester formation, generating a side‐to‐end linkage not previously observed in graspetides. Various efforts to determine the ring connectivity using the previously established tandem mass (MS/MS) analysis of reaction intermediates or partially hydrolyzed products were unsuccessful.^[^ ^12^ ^]^ Collectively, these data suggest that the PIMT homolog‐containing BGC produces a graspetide.

Biochemical analyses suggest that SsfM shares the same enzymatic activity as other RiPP‐associated PIMT homologs. First, we co‐expressed SsfM with SsfA and SsfB, and found that the resulting modified SsfA, named SsfA(BM), and its GluC‐digested fragment, SsfA(BM)_63–97_, had the same molecular weight (MW) as SsfA(B) and SsfA(B)_63–97_, respectively (Figure 2b; Figure S2a, Supporting Information). Unlike other PIMT homolog‐modified RiPPs,^[^ ^11^ ^]^ we could not separate SsfA(BM)_63–97_ and SsfA(B)_63–97_, using HPLC (Figure S2b, Supporting Information). Second, we reconstituted the reaction in vitro with the purified SsfM, the leaderless SsfA(B)_63–97_, and *S*‐adenosylmethionine (SAM). MALDI‐TOF‐MS analyses at multiple time points revealed an initial gain of 14 Da, followed by a loss of 32 Da and a gain of 18 Da, which are consistent with the previously reported reaction pathway including methylation, aspartimidylation, and hydrolysis (Figures 1a and 2c). Third, the isolated intermediates enriched in the aspartyl‐*O*‐methyl ester or aspartimide intermediate (+14 Da or −18 Da from the SsfA(B)_63–97_ MW, respectively) underwent the same MW changes in the absence of SsfM, suggesting that the last two steps, aspartimidylation and hydrolysis, do not require SsfM (Figure S2c, Supporting Information). Fourth, MS and MS/MS analyses of the hydrazine‐added aspartimide intermediate resulted in a 32 Da increase in Asp81, providing another evidence for the presence of the aspartimide intermediate (Figure S3, Supporting Information).^[^ ^17^ ^]^ Finally, SsfM did not efficiently modify partially cyclized SsfA_63–97_ variants containing 0–4 ester linkages or the full‐length unmodified SsfA, indicating that SsfM requires the fully cyclized (fivefold) peptide (Figure S4, Supporting Information). These results are consistent with previous reports,^[^ ^11^ ^]^ suggesting that the RiPP‐associated PIMT homologs generally recognize the cyclized peptide as their substrate.

The multidimensional NMR analyses indicate that SsfM converts Asp81 to a mixture of isoAsp81 and Asp81. We used SsfA(B)_66–97_ and SsfA(BM)_66–97_ enriched with ^13^C and ^15^N to perform 2D ^1^H‐^15^N HSQC, 3D HNCACB,^[^ ^18^ ^]^ 3D HNcoCACB,^[^ ^19^ ^]^ 3D HNCO,^[^ ^20^ ^]^ 3D HNcaCO,^[^ ^21^ ^]^ and 3D hNcocancaNH^[^ ^22^ ^]^ experiments. We assigned the chemical shifts of backbone amide proton (H^N^), nitrogen (N), CO, C^α^, and C^β^ nuclei for all non‐proline residues. We identified seven independent peptide fragments (chains A‐G for SsfA(B)_66–97_; chains A’‐G’ for SsfA(BM)_66–97_), in which chains B, C, and E comprise a single long chain and chains A and D constitute another one (Figure S5 and Supporting Dataset S1, Supporting Information). ^1^H‐^15^N HSQC resonances from a smaller region of chain D’ (Gly77–Thr78) and chain F’ (Asp81–Ser89) were uniquely observed with SsfA(BM)_66–97_, while the others were observed in both molecules (Figure S5, Supporting Information). Close inspections of spectra clearly indicate the presence of isoAsp at the position of Asp81 in the chain F’ of SsfA(BM)_66–97_. First, the phases of C^α^ and C^β^ cross‐peaks were inverted both in the strips taken from the ^1^H^N^─^13^C planes at the nitrogen chemical shifts of Gly82 of HNcoCACB and HNCACB spectra (Figure 2d). Several studies have previously reported these signals as indicative of the isopeptide linkage.^[^ ^23^ ^]^ Second, the HNCO signal of Gly82 was not connected to the HNcaCO signal of Asp81, which indicates that C^β^ is between backbone C^α^ and CO, suggesting that isoAsp has been formed (Figure S6, Supporting Information). We also observed isoAsp at the Asn83 position in chain G of SsfA(B)_66–97_ and in chain G’ of SsfA(BM)_66–97_, suggesting that Asn83 underwent spontaneous aspartimidylation and hydrolysis (Figure S7, Supporting Information).^[^ ^24^ ^]^

### 2.2 RiPP‐Associated PIMT Homologs Share a Conserved C‐Terminal Domain

The common enzymatic activity of PIMT homologs associated with several RiPP subclasses suggests that these enzymes have a close evolutionary relationship. To investigate this further, we used a bioinformatic approach (Figure S8, Supporting Information). Initially, we used SsfM as a single query for the PSI‐BLAST^[^ ^25^ ^]^ to retrieve 73855 PIMTs or their homologs. To simplify the analysis, we reduced the number of proteins to 23490 using a cutoff of 70% sequence identity. We generated a maximum likelihood tree and analyzed their domain architecture as well as gene neighbors. We identified putative BGCs for lanthipeptides, lasso peptides, and graspetides, as well as over 1750 *surE*‐*pcm* clusters in which *pcm* encodes a PIMT homolog not associated with RiPP biosynthesis. This PIMT mediates the isoAsp‐to‐Asp conversion in abnormal proteins in which isoAsp spontaneously arises from Asp and Asn residues, and enhances *E. coli* survival under stress conditions in the late stationary phase.^[^ ^26^ ^]^

Notably, we observed that the majority of putative RiPP BGCs were contained in a single clade of 4003 enzymes, while the *surE*‐*pcm* clusters were predominantly located outside of this clade (Figure S8, Supporting Information). Additionally, in this clade, 3200 PIMT homologs (80%) possessed a C‐terminal extension of over 100 amino acids (Figure S8, Supporting Information). This C‐terminal domain was also identified in PIMT homologs for lanthipeptides, lasso peptides, and graspetides (OlvS, TceM, and AmdM, respectively).^[^ ^11a,b,d^ ^]^ A PIMT enzyme from *Thermotoga maritima* (*Tm*PIMT) is, to our knowledge, the only enzyme in this clade with an experimentally determined 3D structure (PDB 1DL5).^[^ ^27^ ^]^ By comparing this structure and several predicted structures, we found that the C‐terminal domains in this clade are highly homologous. First, we obtained the predicted structures of full‐length SsfM, OlvS, and TceM using AlphaFold^[^ ^28^ ^]^ (Figure S9a, Supporting Information). To avoid the potential bias from using the structure of *Tm*PIMT as the template, we also obtained the predicted structures of their C‐terminal domains using ColabFold^[^ ^29^ ^]^ (Figure S9b, Supporting Information) and those of full‐length enzymes using a template‐independent ESMFold^[^ ^30^ ^]^ (Figure S9c, Supporting Information). For each enzyme, the structures of the C‐terminal domain in the latter two models were highly homologous to the one in the full‐length AlphaFold model (R.M.S.D. ≤1 Å). All structures have the same arrangement of secondary structures, βαββββα, as that in *Tm*PIMT (Figures S9a–c, Supporting Information). The pairwise structural alignments of the domains in the AlphaFold structure on the Dali server^[^ ^31^ ^]^ revealed, albeit weak due to shifts of secondary structures, similarity between these regions with a *Z*‐score of 3.4–7.3 (Figure S9d, Supporting Information). These findings suggest that this domain is conserved among RiPP‐associated PIMT homologs and may have a potential role in RiPP biosynthesis. The latter has been recently proposed in a biochemical analysis of AmdM involved in the maturation of a graspetide, amycolimiditide.^[^ ^11d^ ^]^ We suggest renaming these RiPP‐associated PIMT homologs as peptide/protein L‐aspartyl *O*‐methyltransferases or PAMTs, given that they primarily modify L‐aspartate as a natural substrate while they may also accept isoaspartate as well.

### 2.3 Genome Mining of PAMTs Reveals a Novel RiPP Subclass

**Figure 3:**
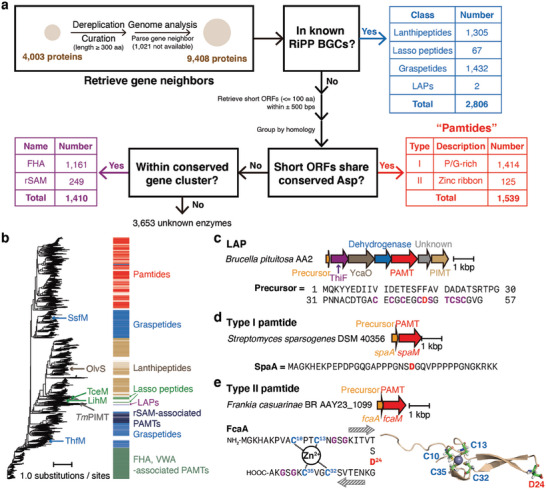
PAMTs are involved in the biosynthesis of various RiPPs. a) A schematic workflow of the genome mining of PAMTs containing C‐terminal domains. b) A maximum likelihood tree of 9408 enzymes is generated, and biochemically characterized PAMTs are annotated. PAMTs encoded in either known RiPP BGCs (graspetides, blue; lanthipeptides, brown; lasso peptides, green; LAPs, purple), newly identified RiPP BGCs (pamtides, red), or conserved gene clusters (FHA‐VWA, dark green; rSAM, dark blue) are labeled with color strips. c–e) Model BGCs for LAPs (c), type I pamtides (d; SpaA, precursor; SpaM, PAMT) and type II pamtides (e; FcaA, precursor; FcaM, PAMT). Sequences of precursors (SpaA and FcaA) are shown below each gene cluster. The structure of FcaA was predicted by Alphafold and zinc ion was modeled by MIB. Conserved residues in FcaA are highlighted by colors (cysteine, blue; glycine, purple; aspartate, red).

High sequence homology, the conserved C‐terminal domain, and the common enzymatic reaction suggest that PAMTs have evolved from a common ancestor. Furthermore, their frequent association with RiPP biosynthesis implies that this ancestral PAMT has spread to multiple unrelated RiPP subclasses. Therefore, we hypothesized that further exploration of this clade could uncover novel RiPP subclasses that utilize PAMTs as either primary or secondary modification enzymes. To test this idea, we compiled the expanded list of PAMTs in this clade without the cutoff of 70% sequence identity and eliminated proteins that are either shorter than 300 amino acids or devoid of genomic information for neighboring genes, resulting in 9408 enzymes (**Figure** **3**a). Analysis of gene neighbors for known RiPP biosynthetic enzymes or precursor peptides revealed additional BGCs for linear azol(in)e‐containing peptides (LAPs; 2 BGCs) as well as lanthipeptides (1305 BGCs), lasso peptides (67 BGCs), and graspetides (1432 BGCs), of which the numbers of BGCs for the latter three increased 5–50% from previous reports (lanthipeptides, 837; lasso peptides, 48; graspetides, 1326; Figure 3a,b; Supporting Dataset S1, Supporting Information).^[^ ^11^, ^13^, ^32^ ^]^ Consistent with recent comprehensive genome mining of lanthipeptides,^[^ ^32^ ^]^ PAMTs in lanthipeptide BGCs are associated with class I lanthipeptides and most precursor peptides adopt the TxDGC core motif (Figure S10a, Supporting Information). Precursors for lasso peptides can be classified into two groups based on core motifs; one group contains the DTAD motif in the lasso ring as previously reported,^[^ ^11b^ ^]^ while the other group shares a highly conserved Asp residue in the putative lasso loop (Figure S10b, Supporting Information). Precursors encoded in two LAP BGCs have an Asp residue within the C/S/T/G‐rich core motif (Figure 3c; Figure S10c, Supporting Information).^[^ ^1^, ^33^ ^]^ In total, we assigned 2806 of 9408 PAMTs into four known subclasses of RiPPs.

Nonetheless, most enzymes (70.2%) did not show any obvious association with known RiPP BGCs. We hypothesized that some of these enzymes could be involved in the biosynthesis of new RiPP subclasses. Indeed, we found a large number of two‐gene clusters encoding a putative precursor peptide and a PAMT (1183 non‐redundant putative precursors associated with 1539 non‐redundant PAMTs), but no primary modification enzymes for known RiPPs. Analysis of putative precursor peptides revealed two major types with distinct sequence features. Type I precursors have ≈45 amino acids and are rich in Gly (17.3%), Pro (16.8%), and Asp (10.2%; Figure 3d; Figure S11a, Supporting Information). They present several different conservation patterns of the sequences but commonly have at least one highly conserved Asp residue nearby conserved prolines or glycines. By contrast, type II precursors contain a highly conserved zinc ribbon motif commonly found in DnaJ (PF00684) with a conserved Asp at the center (CxxCxGxG_D_CxxCxGxG; Figure 3e; Figure S11b, Supporting Information).^[^ ^34^ ^]^ Four conserved cysteines in the zinc ribbon motif coordinate a zinc ion and the intervening residues form two anti‐parallel β‐strands (Figure S12, Supporting Information). The predicted structure of a type II precursor by AlphaFold and a metal ion‐binding site prediction server (MIB) also showed the typical zinc ribbon, in which the conserved Asp residue is located in the hairpin (Figure 3e).^[^ ^28^, ^35^ ^]^ The two‐gene architecture with a highly homologous PAMT enzyme and the presence of a conserved Asp residue in putative precursors suggest that these PAMTs serve as a primary modification enzyme for the Asp derivatization in the putative precursor, defining a novel subclass of RiPPs. We propose the name “pamtides” for those produced by these distinct BGCs.

We also identified 1410 PAMTs associated with the conserved gene clusters that typically contain ten genes as well as ABC transporter genes (Figure S13a, Supporting Information). In particular, the PAMT gene is located next to a gene encoding forkhead‐associated (FHA) domain‐containing protein. This protein contains a long N‐terminal Pro/Gly‐rich region with a few Asp residues, similar to the putative precursors for type I pamtides, suggesting that PAMT in this gene cluster may modify the FHA domain‐containing protein.

We also observed an additional 249 distinct gene clusters consisting of two genes encoding a radical *S*‐adenosylmethionine (rSAM) enzyme and a PAMT. However, we could not find any neighboring genes encoding putative precursors or substrate proteins for modification (Figure S13b, Supporting Information). Although we could not obtain any clues that the remaining 3653 enzymes are associated with RiPP biosynthesis or protein PTM, we cannot exclude the possibility that these enzymes are also involved in the same type of modification reactions.

### 2.4 A PAMT Enzyme in Type II Pamtide Biosynthesis Mediate the Asp‐to‐isoAsp Conversion

**Figure 4:**
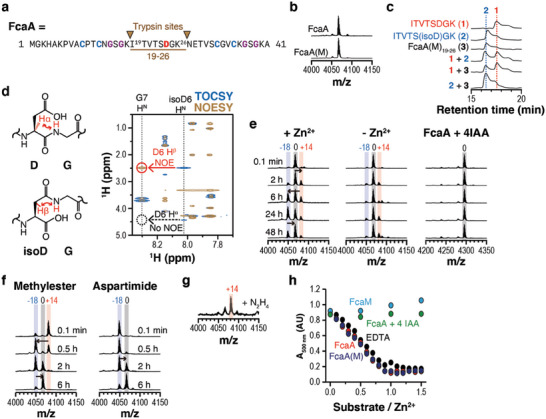
FcaM mediates isoaspartate installation in a type II pamtide. a) Trypsin cleavage sites in FcaA. b) MALDI‐TOF‐MS spectra of the purified recombinant FcaA and FcaA(M). c) HPLC analysis of FcaA(M)_19–26_ (**3**) with synthetic peptides **1** (ITVTSDGK) and **2** (ITVTSisoDGK). d) NMR analysis of FcaA(M)_19–26_. The NOE signal indicates that Asp24 is converted to isoaspartate. e) MALDI‐TOF‐MS spectra of FcaM‐mediated modification of FcaA or its variant in vitro. FcaA or iodoacetamide‐labeled FcaA (FcaA+4IAA, 50 µm) was mixed with FcaM (10 µm) in the presence of DTT (1 mm), Tris‐HCl pH 8.0 (20 mm), and ZnCl_2_ (0 or 100 µm) at 25 °C. Reactions were monitored at designated time points by mass analyzer. f) MALDI‐TOF‐MS spectra showing spontaneous chemical transformation of methylester‐ (left) or aspartimide‐containing intermediates (right). Peptides (20‐100 µM) were dissolved in a buffer containing Tris‐HCl (20 mm, pH 8.0), DTT (1 mm), and ZnCl_2_ (100 µm). The mixture was incubated at 25 °C for designated time points. g) MALDI‐TOF‐MS spectrum of the hydrazide‐containing peptide. The reaction condition for Figure S3a (Supporting Information) was adopted to trap the aspartimide by hydrazine. h) Competition assay of 4‐(2‐pyridylazo)resorcinol (PAR) and various substrates toward Zn^2+^. ZnCl_2_ (10 µm) was mixed with PAR (100 µm) in a buffer containing Tris‐HCl (20 mm, pH 8.0) and NaCl (100 mm). 0–15  µm substrates were added to the mixture and absorbance at 500 nm was monitored in each mixture. Each data point is colored based on the substrate. Chemical shift values, observed, and calculated mass values can be found in Supporting Dataset S1 (Supporting Information).

To test whether PAMTs in the pamtide BGCs convert Asp to aspartimide or/and isoaspartate in the precursor peptides, we initially selected one BGC for type II pamtide from *Frankia cauarinae* BR AAY23_1099 (Figures 3e and **4**a). Heterologous co‐expression of the precursor (FcaA) and PAMT (FcaM) in *E. coli* showed that the product, FcaA(M), had the same MW as the unmodified FcaA (Figure 4b). We purified FcaA(M) and digested it with trypsin to obtain FcaA(M)_19–26_ (**3**; Figure 4c) containing the conserved Asp residue. We also chemically synthesized the FcaA_19–26_ equivalent (ITVTSDGK, **1**) and its isoAsp variant (ITVTS(isoD)GK, **2**). The comparison of HPLC chromatograms of individual peptides or their combinations revealed that the major component of FcaA(M)_19–26_ is equivalent to the isoAsp variant and clearly different from FcaA_19–26_ (Figure 4c).

We also obtained the ^1^H, ^1^H‐^1^H COSY, ^1^H‐^1^H TOCSY, and ^1^H‐^1^H NOESY spectra for the three peptides and assigned the chemical shifts of protons. In the NOESY spectrum of FcaA(M)_19‐26_, we observed a NOE signal between G25 H^N^ and D24 H^β^, but not between G25 H^N^ and D24 H^α^ (Figure 4d; Figure S14 and Supporting Dataset S1, Supporting Information), which is consistent with the previous observation for OlvA(BCS_A_)^GluC^, an isoAsp‐containing lanthipeptide.^[^ ^11a^ ^]^ The chemically synthesized ITVTS(isoD)GK (**2**) also showed this correlation, but the unmodified peptide ITVTSDGK (**1**) presented the reverse correlation (Figure S15, Supporting Information). These analyses consistently support that FcaM mediates the Asp‐to‐isoAsp conversion in FcaA.

To characterize the FcaM‐mediated reaction in detail, we reconstituted the reaction in vitro under various conditions (Figure 4e). To prevent the formation of disulfide bonds, we provided 1,4‐dithiothreitol (DTT) in the reaction solutions. In the presence of Zn^2+^, FcaA displayed the same MW changes as those of SsfA(B)_63‐97_ and other cyclized intermediates of RiPPs associated with PAMT enzymes: an initial gain of 14 Da, followed by a loss of 32 Da and a gain of 18 Da (Figure 4e). The isolated intermediates also showed spontaneous conversions to the species with the original MW and hydrazine trapping of the putative aspartimide species (−18 Da) resulted in the hydrazine‐added species (+14 Da; Figure 4f,g). However, no MW changes were observed when Zn^2+^ was absent or cysteines in FcaA were blocked by iodoacetamide (IAA), indicating that coordination of cysteines in FcaA to Zn^2+^ is essential for the modification. Additionally, the complex of Zn^2+^ and 4‐(2‐pyridylazo)resorcinol (PAR),^[^ ^36^ ^]^ a metal‐sensitive colorimetric reagent, was completely dissociated by adding equal amount of FcaA or FcaA(M), but not by FcaM or IAA‐labeled FcaA, suggesting that Zn^2+^ binds to FcaA or FcaA(M) in a 1:1 ratio (Figure 4h). Although we are not entirely confident that the endogenous metal ion would be Zn^2+^, our bioinformatic analysis and in vitro reconstitution strongly suggest that, in line with other PAMT‐associated RiPPs, the type II pamtides require the “cyclic” or “hairpin‐like” architecture for the Asp‐to‐isoAsp conversion and metal–ligand interactions can be a strategy for the formation of cyclic architecture.

### 2.5 A PAMT Enzyme in Type I Pamtide Biosynthesis Catalyzes the Similar Conversion in the Precursor Peptide

**Figure 5:**
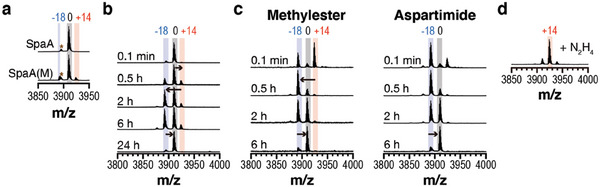
SpaM for a type I pamtide catalyzes the homologous reaction to the linear peptide, SpaA. a) MALDI‐TOF‐MS spectra of the purified recombinant SpaA (top) or SpaA(M) (bottom). Brown asterisks denote the laser‐induced deamination of 0 Da species in the mass analyzer. b) MALDI‐TOF‐MS spectra of SpaM‐mediated SpaA modification in vitro. SpaA (20 µm) was mixed with SpaM (5 µm) in presence of DTT (1 mm), SAM (1 mm), and Tris‐HCl pH 8.0 (50 mm) at 25 °C. Reaction was monitored at designated time points by mass analyzer. Relative mass value changes to the intact SpaA are given above peaks. c) MALDI‐TOF‐MS spectra of spontaneous rearrangement of methylester‐ (left) or aspartimide‐containing intermediates (right). Reaction conditions for Figure S2c (Supporting Information) were adopted to observe the spontaneous rearrangement of the intermediates. d) MALDI‐TOF‐MS spectrum of hydrazide‐containing peptide. Same reaction condition as Figure S3a (Supporting Information) was applied to the aspartimide‐containing intermediate to generate hydrazide. The relative mass value to the intact SpaA is given above the peak. Calculated and observed mass values can be found in Supporting Dataset S1 (Supporting Information).

We also tested a model precursor (SpaA) and a PAMT (SpaM) for type I pamtides from *Streptomyces sparsogenes* DSM 40 356 (Figure 3d). Co‐expression of SpaM and SpaA yielded the species without MW change from intact SpaA as a major product, while those with −18 and +14 Da MW change were also observed (**Figure** **5**a). We additionally found that the SpaM‐mediated reaction displayed almost the same features as those of PAMTs in RiPP biosynthesis: the same pattern of MW changes at the conserved aspartate in the in vitro reaction, the spontaneous conversions of the reaction intermediates, and the hydrazine trapping of the aspartimide intermediate (Figure 5b–d; Figure S16, Supporting Information). Although we could not directly confirm the Asp‐to‐isoAsp conversion with SpaM, the same reaction features and the evolutionary relationship of SpaM with other PAMT enzymes suggest that SpaM most likely mediates the same modification reaction.

Notably, BGCs for type I pamtides do not encode a conserved cyclase and precursors do not present known intrinsically cyclic or hairpin‐like motifs. Additionally, AlphaFold‐predicted structures of SpaA and SpaA‐SpaM complex do not present the cyclic architecture within SpaA (Figure S17a, Supporting Information). Most precursors contain highly conserved acidic/basic residues at their N‐/C‐termini (Figure S11a, Supporting Information), and the substitution of the conserved C‐terminal region to alanines was deleterious for the SpaM activity (Figure S17b, Supporting Information). We suggest that these conserved C‐terminal residues might play a critical role in either recognition of PAMT or formation of a cyclic/hairpin structure. Collectively, these data suggest that PAMTs in the pamtide BGCs serve as a primary modification enzyme by mediating the same chemical conversion as other RiPP‐associated PAMT enzymes.

## 3 Conclusion

Overall, we report that highly homologous PAMTs have evolutionarily spread across multiple RiPP subclasses and mediate the same modification reaction that converts between Asp and isoAsp via aspartyl‐*O*‐methyl ester and aspartimide intermediates. More importantly, we show that this evolutionary feature could guide the identification of a novel RiPP subclass, pamtides, in which the PAMT‐mediated Asp‐to‐isoAsp conversion is the primary modification reaction. Using various biochemical characterizations including heterologous co‐expression, in vitro reconstitution, time‐course experiment, and hydrazine trapping as well as mass spectrometry and NMR analyses, we confirmed the conserved PAMT‐mediated Asp‐to‐isoAsp conversion for a group 13 graspetide and a type II pamtide. We also showed the similar reaction features in a type I pamtide. We identified more than 4300 putative RiPP BGCs encoding a PAMT enzyme by mining bacterial genomes. Currently, PAMT‐associated subclasses of RiPPs include pamtides, graspetides, lanthipeptides, lasso peptides, and LAPs. Among them, pamtides are the only subclass in which PAMT functions as a class‐defining modification enzyme.

The functional role of the Asp‐to‐isoAsp conversion is largely unknown. One possibility is that the conversion to isoAsp generates a β‐amino acid that has one additional hydrocarbon in the peptide backbone, thus releasing the ring strain in the macrocyclic structures. Indeed, the majority of the characterized PAMTs modify an Asp residue located in the macrocyclic (e.g., graspetides, lanthipeptides, and lasso peptides) or hairpin‐like (type II pamtides) region of precursor peptides, requiring fully cyclized peptides as substrates.^[^ ^11^ ^]^ Another non‐exclusive possibility is that this conversion simply changes the structure in the loop or hairpin region, thus diversifying the physical or functional properties of RiPPs.^[^ ^11a^ ^]^ Alternatively, as previously suggested, the electrophilic aspartimide intermediate might be the functional product.^[^ ^11^ ^]^ The microcin C7 biosynthetic pathway was previously reported to involve the formation of an aspartimide intermediate, which is subsequently linked to adenosine monophosphate (AMP) through a P‐N bond and hydrolyzed to produce the Asp‐NH‐AMP moiety within microcin C7.^[^ ^37^ ^]^

The widespread distribution of an accessory protein in multiple RiPP subclasses was also illustrated by the RiPP precursor peptide recognition element (RRE) domain,^[^ ^38^ ^]^ which has not been found in PAMTs. The recent RRE‐guided bioinformatic analysis revealed a novel RiPP class termed daptides.^[^ ^39^ ^]^ It is highly probable that many other proteins with secondary roles in RiPP biosynthesis are also evolutionarily disseminated to unrelated RiPP subclasses. Tailoring enzymes are often shared by distinct classes of natural products,^[^ ^40^ ^]^ and genome mining of a tailoring enzyme was recently applied to discover unprecedented fungal arginine‐containing cyclodipeptides.^[^ ^41^ ^]^ Given that the genome mining focused on a class‐defining modification enzyme typically expands the members of the same RiPP subclass, we believe that genome mining of accessory proteins is a powerful approach to identify novel RiPP subclasses and unprecedented PTMs.

## Conflict of Interest

The authors declare no conflict of interest.

## Supporting information

Supporting Information

Supporting Information

## Data Availability

The data that support the findings of this study are available in the supplementary material of this article.
