# Supplementary material for: Evolutionary Spread of Distinct O‐methyltransferases Guides the Discovery of Unique Isoaspartate‐Containing Peptides, Pamtides
Source: Adv Sci (Weinh). 2023 Nov 20;11(2):2305946. doi: 10.1002/advs.202305946 (PMC10787088; doi:10.1002/advs.202305946)
Supplement: Supplementary file 1 — Supporting Information [file ADVS-11-2305946-s001.pdf]

ADVANCED SCIENCE
Open Access

Supporting Information

for *Adv. Sci.*, DOI 10.1002/advs.202305946

Evolutionary Spread of Distinct *O*-methyltransferases Guides the Discovery of Unique Isoaspartate-Containing Peptides, Pamtides

*Hyunbin Lee, Sho Hee Park, Jiyoon Kim, Jaehak Lee, Min Sun Koh, Jung Ho Lee and Seokhee Kim**

Supporting Information

Evolutionary spread of distinct *O*-methyltransferases guides the discovery of unique isoaspartate-containing peptides, pamtides

*Hyunbin Lee, Sho Hee Park, Jiyoon Kim, Jaehak Lee, Min Sun Koh, Jung Ho Lee, and Seokhee Kim**

Table of Contents

Experimental Procedures **S3**

General materials and methods S3
Cloning S3
Heterologous expression and protein purification S3
Mass analysis of SsfA(BM), FcaA(M), and SpaA(M) S4
Size exclusion chromatography S4
Proteolytic digestion S4
Iodoacetamide labeling of FcaA S4
High performance liquid chromatography (HPLC). S4
In vitro reconstitution of enzymatic and chemical reactions S5
Solid phase peptide synthesis S5
Assignment of protein backbone chemical shifts of SsfA(BM)$_{66–97}$ and SsfA(B)$_{66–97}$ S5
Sequential assignment of proline residues in SsfA(B)$_{66–97}$ S6
NMR analysis of synthetic peptides and FcaA(M)$_{19–26}$ S6
Genome mining of pamtides S6
Protein structure prediction and analysis S7
Metal binding assay S7

Supplementary Figures **S8**

Figure S1. Mass analysis of the methanolyzed SsfA(B)$_{63–97}$ S8
Figure S2. MS and LC analysis of SsfA(B) and SsfA(BM) variants. S9
Figure S3. Mass analysis of hydrazide-containing SsfA(BM)$_{63–97}$ S10
Figure S4. MALDI-TOF-MS spectra of SsfM-mediated modification reaction of SsfA variants S11
Figure S5. Assigned Chains and ^{1}H-^{15}N HSQC spectra of SsfA(B)$_{66–97}$ and SsfA(BM)$_{66–97}$ S12
Figure S6. HNcaCO and HNCO analysis of SsfA(BM)$_{66–97}$ chain E'/F' S14
Figure S7. HNCACB, HNcoCACB, HNCO, and HNcaCO analysis of SsfA(BM)$_{66–97}$ chain G' S15
Figure S8. Bioinformatic analysis workflow of PIMTs and RiPP-associated PIMT homologs S16
Figure S9. Structures of the C-terminal domains in model RiPP-associated PIMT homologs S17
Figure S10. Features of PAMT-associated RiPPs S19
Figure S11. Sequence logos of precursor peptides for pamtides S20
Figure S12. Structure of zinc ribbon S21
Figure S13. Conserved PAMT-associated gene clusters S22
Figure S14. NMR spectra of FcaA(M)$_{19–26}$ S23
Figure S15. NMR analysis of synthetic ITVTSDGK and ITVTS(isoD)GK S25
Figure S16. MS/MS analysis of SpaA derivatives S30
Figure S17. Model structure of SpaA and MS analysis of its variants S31

References **S32**

Author contributions **S33**

Experimental Procedures

General materials and methods. Genes optimized for heterologous expression of recombinant precursor peptides, ATP-grasp enzymes, and peptide/protein L-aspartyl *O*-methyltransferases (PAMTs) in *Escherichia coli* (*E. coli*) were synthesized by Gene Universals (USA, Supplementary Dataset 1). Oligonucleotides were purchased from Bionics (Korea). All reagents for cloning were purchased from Enzynomics (Korea) unless otherwise specified. *E. coli* ER2566 (DE3) was used for cloning and protein production. Appropriate antibiotics were added to the media and LB-agar plates to a final concentration of 100 μg/mL. Protein concentrations were determined by UV absorbance at 280 nm or 214 nm. Amicon centrifugal filters were purchased from Merck Millipore (USA). Trypsin and chymotrypsin were purchased from Sigma-Aldrich (USA), and endoproteinase GluC was purchased from NEB (USA). For mass analysis, samples were desalted by C18 ZipTip (Merck, USA) if needed, and Bruker UltraFlextreme MALDI-TOF/TOF-MS was used for the analysis (Bruker Daltonics, USA). $^{13}C_6$-d-glucose and $^{15}NH_4Cl$ were purchased from Cambridge Isotopes Laboratorie (USA). Fmoc-protected isoaspartate (Fmoc-Asp-Otbu) was purchased from Alfa Aeser (USA) and the rest of Fmoc-protected amino acids and Wang resin for solid phase synthesis were purchased from GL Biochem (China) unless otherwise noted.

Cloning. Plasmids and primers used in this study are listed in Supplementary Dataset 1. SsfB gene in the dicistronic plasmid pHB665 was deleted by inverse-PCR mutagenesis[1] to obtain a plasmid encoding recombinant SsfA. *E.coli* maltose-binding protein (MBP) and TEV protease cleavage site ([TEV]) were fused to the N-terminus of FcaA and SpaA by overlap-extension PCR.[2] In brief, F_22b and R_TEV were used as primers to generate the His_6-MBP-[TEV] fragment, and R_22b and F_XxxA (XxxA = FcaA or SpaA) were used to yield XxxA fragment at the first PCR step. Two fragments were used as template, and R_TEV and F_XxxA were used as primers of the second PCR step.

PCR products were purified by LaboPass PCR Purification Kit (Cosmogenetech, Korea) following the instructor's guide. Purified products were phosphorylated and ligated using T4 Polynucleotide kinase and T4 DNA ligase). Ligated DNA products were transformed into *E. coli*. Cells were grown on an LB-agar plate containing corresponding antibiotics at 37 °C for 12–16 hours. Each colony was inoculated in 2 mL of LB with antibiotics at 37 °C for 12–16 hours, and plasmids were purified using LaboPass Plasmid Miniprep Kit (Cosmogenetech, Korea). Sequences were verified by Sanger sequencing method (Bionics, Korea).

Heterologous expression and protein purification. Plasmids encoding desired proteins were transformed into E. coli. Cells were grown on an LB-agar plate with appropriate antibiotics at 37 °C for 12-16 hours. A single colony was inoculated in 10–80 mL of LB containing antibiotics and grown at 37 °C for 12–16 hours. The culture was 100-fold diluted in 1–8 L of fresh LB with antibiotics and further inoculated at 37 °C. When OD_{600} reached between 0.4 and 0.6, protein production was induced by adding β-D-1-thiogalactopyranoside (IPTG; LPS solutions, Korea) to a final concentration of 0.1 mM. Cells were incubated at 25 °C for 16–24 hours and then they were harvested by centrifugation at 5,200 xg for 15 minutes. Cell pellets were stored at -80 °C for less than four days before use. To obtain the ^{13}C- and ^{15}N-labeled SsfA(B) or SsfA(BC), 1 L of M9 media with the following components were prepared for protein production: $^{15}NH_4Cl$ (1.5 g; Cambridge Isotopes Laboratories), ^{13}C glucose (2.0 g; Cambridge Isotopes Laboratories), Na_2HPO_4 (6.0 g; LPS solutions), KH_2PO_4 (3.0 g; LPS solutions), NaCl (0.5 g; LPS solutions), ISOGRO-^{13}C, ^{15}N powder (0.5 g; Sigma-Aldrich), 100X MEM vitamin solution (5 mL; ThermoFisher, USA), $MgCl_2$ (1 mM at final; LPS solutions), $CaCl_2$ (0.1 mM at final; LPS solutions), and 1,000X trace metal solution (24.7 mM $FeCl_3$, 0.76 mM $CuCl_2$, 0.05 mM $MnCl_2$, 0.77 mM $CoCl_2$, H_3BO_3 1.6 mM, and $ZnCl_2$ 6.16 mM; 1 mL). Single colony harboring pHB665 or both pHB665 and pHB666 were inoculated in 50 mL of LB containing ampicillin or both ampicillin and streptomycin at 37 °C for 16 hours. When OD_{600} was reached around 3.0–4.0, cells were harvested and transferred into 1 L of the prepared M9 media to have an initial OD_{600} around 0.2. Cells were grown at 37 °C until OD_{600} reached

0.5–0.6. Protein production was induced by adding IPTG to a final concentration of 0.1 mM. Cells were further incubated at 25 °C for 24 hours and harvested by centrifugation.

Cell pellets were resuspended in 20–160 mL of wash buffer (50 mM Tris-HCl pH 8.0, 20 mM imidazole, 300 mM NaCl) and lysed by sonication. Insoluble fractions were eliminated by centrifugation at 18,000 xg for 30 minutes at 4 °C. Supernatants were applied to 1–5 mL of Ni resin pre-equilibrated with wash buffer (Ni Sepharose 6 Fast Flow beads, GE Healthcare, USA). Resins were washed twice with a volume of wash buffer equal to 5 column volumes. Proteins were eluted by adding 3 column volume of elution buffer (50 mM Tris-HCl pH 8.0, 500 mM imidazole, 100 mM NaCl). Fractions were concentrated and the buffer was exchanged to buffer A (10 mM Tris-HCl pH 8.0, 100 mM NaCl) using Amicon centrifugal filter. Enzymes were further purified by size exclusion chromatography, and precursor peptides were digested by appropriate proteases and purified by High performance liquid chromatography (HPLC).

Mass analysis of SsfA(BM), FcaA(M), and SpaA(M). For the production of mass spectrum in Figure 2b, eluted SsfA(BM) was immediately desalted by C18 ZipTip and subjected to mass analysis to prevent potential spontaneous derivatization of methylester- or aspartimide-containing variants. Similarly, to produce mass spectra in Figures 4b and 5a, TEV protease (40 μM) and DTT (1 mM) were immediately added to the eluted fractions containing recombinant FcaA(M) and SpaA(M). The mixtures were incubated at 25 °C for 1 hour, desalted by C18 ZipTip, and subjected to MALDI-TOF/TOF-MS.

Size exclusion chromatography. Size exclusion chromatography was performed using ÄKTA pure 25M1 (GE Healthcare, USA) on a Superdex200 10/300 GL column (Cytiva, USA) equilibrated in buffer A. Fractions were monitored by UV absorbance at 280 nm and SDS-PAGE. Desired fractions were collected and concentrated using Amicon centrifugal filter, and stored at -80 °C.

Proteolytic digestion. To eliminate SsfB (and SsfC) from SsfA(B) (or SsfA(BC)), trifluoroacetic acid was added to purified SsfA(B) + SsfB (or SsfA(BC) + SsfB + SsfC) to a final concentration of 1 % (v/v), and 1 M Tris-HCl pH 8.0 was added to the mixture to neutralize pH up to 7.0–8.0. Precipitated enzymes were eliminated by centrifugation at 20,000 xg for 10 minutes at 4 °C. Trypsin or GluC was added to the supernatant containing SsfA derivatives (1/100 of SsfA derivatives by weight), and incubated at 37 °C for 16 hours. Cleavage reaction was monitored by MALDI-TOF-MS, and desired fragments were further purified by HPLC.

MBP-fused precursor peptide derivatives (1 mM; FcaA, FcaA(M), SpaA, or SpaA(M)) were digested by adding 10 μM of TEV protease, 1 mM dithiothreitol (DTT), and 20 mM Tris-HCl pH 8.0. The mixtures were incubated at 25 °C for 16 hours. MBP was eliminated by an Amicon centrifugal filter, and the peptides were further purified by HPLC to fully eliminate bound zinc ions from FcaA variants. To yield $FcaA(M)_{19–26}$, trypsin was added to the HPLC-purified FcaA(M) (1/10 by weight) and reaction mixture was incubated at 37 °C for 24–48 hours. The reaction was monitored by MALDI-TOF-MS and purified by HPLC.

Iodoacetamide labeling of FcaA. The following mixture was prepared to alkylate cysteines in FcaA: 100 μM FcaA, 1 mM iodoacetamide, 1 mM TCEP, 20 mM Bicine pH 8.5. Reaction mixture was incubated at 37 °C for 2 hours. Reactions were monitored by MALDI-TOF-MS and purified by HPLC.

High performance liquid chromatography (HPLC). HPLC was performed using Agilent 1260 Infinity (Agilent, USA). A ZORBAX SB-C18 analytical column (4.6 x 250 mm, particle size 5 μm, Agilent, USA) or a ZORBAX SB-C18 Semi-preparative column (9.4 x 250 mm, particle size 5 μm, Agilent, USA) was used for the LC analysis or purification, respectively. Two mobile phases were used in HPLC: Solvent A (0.05 % Trifluoroacetic acid in H_2O, v/v) and solvent B (0.05 % Trifluoroacetic acid in CH_3CN, v/v). Peptides were separated by linearly increasing the concentration of CH_3CN. Fractions were monitored by

UV absorbance at 214 nm or 280 nm, and MALDI-TOF-MS. Desired fractions were collected and freeze-dried. Dried peptides were dissolved in milli-Q H2O immediately before use.

***In vitro* reconstitution of enzymatic and chemical reactions.** SpaM- or SsfM-mediated modification reaction of SpaA or SsfA variants were reconstituted as follows: 20 μM of substrate, 5 μM of enzyme, 1 mM of *S*-adenosylmethionine (SAM), 1 mM of DTT, and 50 mM of Tris-HCl pH 8.0. FcaM-mediated reactions of FcaA variants were reconstituted as follows: 100 μM of substrate were mixed with FcaM (20 μM) in presence of DTT (1 mM), Tris-HCl pH 8.0 (20 mM), and $ZnCl_2$ (100 μM). All reaction mixtures were incubated at 25 °C and monitored by MALDI-TOF-MS at designated time points. Reaction mixtures were subjected to HPLC to purify the observed intermediates.

To observe the spontaneous transformation of aspartyl-*O*-methylester to aspartimide and aspartimide to aspartate/isoaspartate, peptides containing aspartyl methylester or aspartimide were dissolved in either 10 mM Tris pH 8.0 (SsfA or SpaA derivatives) or 20 mM Tris pH 8.0, 1 mM DTT, and 100 μM $ZnCl_2$ (FcaA derivatives) to the final concentrations of 10–100 μM. All reaction mixtures were incubated at 25 °C and monitored by MALDI-TOF-MS at designated time points.

For the generation of aspartyl hydrazide, aspartimide-containing peptides were dissolved in 2 M hydrazine pH 8.0 to the final concentrations of 20–100 μM. Solutions were incubated at 37 °C for 2 hours. Reactions were monitored by MALDI-TOF-MS and MALDI-TOF/TOF-MS, if needed. For the SsfA derivative, NaOH was added to a final concentration of 0.1 M to hydrolyze ester bonds.

Solid phase peptide synthesis. Peptides (ITVTSDGK, **1**; ITVTS(isoD)GK, **2**) were synthesized as previously described with modifications[3]. First amino acid was coupled as follows: Wang resin was mixed with Fmoc-protected lysine (5 equiv.) and 4-Dimethylaminopyridine (DMAP, 0.1 equiv.) in *N*,*N*-dimethylformamide (DMF). *N*,*N*'-Diisopropylcarbodiimide (DIC, 4 equiv.) was added to the mixture after 20 minutes. The mixture was further incubated at room temperature for 40 hours.

Remaining amino acids were coupled as follows: 20% piperidine in DMF (v/v) was added to the resins to remove Fmoc groups. Mixtures were incubated at room temperature for 1 hour. Fmoc-protected amino acid (5 equiv.) was dissolved in DMF containing HATU (5 equiv.) and *N*,*N*'-Diisopropylethylamine (DIPEA, 10 equiv.) and mixed with the resins to attach the amino acid. The mixture was incubated at room temperature for 1 hour. Resins were washed with DMF and dichloromethane three times between each step, and each reaction was incubated under rigorous shaking. The coupling reaction was finished by removing Fmoc group.

Cleavage cocktail (TFA:H_2O:Triisopropylsilane = 950:25:25, v/v) was added to the resins to detach peptides and remove the remaining protecting groups. The mixture was incubated for 2 hours. Peptides were concentrated by evaporating TFA through air-drying. Ice-cooled ether/hexane (1:1, v/v) was added to the solution to precipitate peptides. Pellets were dried at 50 °C, dissolved in DMSO, and purified by HPLC.

Assignment of protein backbone chemical shifts of SsfA(BM)$_{66–97}$ and SsfA(B)$_{66–97}$. All NMR experiments were acquired using a Bruker AVANCE NEO 600 MHz NMR spectrometer equipped with a 5 mm triple-resonance z-gradient cryogenic probehead. 0.3 mM ^{13}C, ^{15}N-labeled SsfA(BM)$_{66-97}$ and 0.8 mM ^{13}C, ^{15}N-labeled SsfA(B)$_{66-97}$ were separately prepared in the 20 mM sodium phosphate buffer (pH 6.0) containing 20 mM sodium chloride and 5% D_2O and placed in a 5 mm D_2O-matched Shigemi tube.

Backbone assignments of SsfA(BM)$_{66-97}$ at 277.1 K were carried out by performing 3D HNCACB[4], HNcoCACB[5], 3D HNCO[6], and 3D HNcaCO[7] experiments using the BEST scheme[8]. Small case letters indicate nuclei whose chemical shifts were not recorded. 3D HNCACB data was acquired using a data matrix of 120 (t_1, ^{13}C) × 290 (t_2, ^{15}N) × 1024 (t_3, H^N) complex points and sweep widths of 10000 (^{13}C), 1176 (^{15}N), and 6250 (H^N) Hz. The total experiment time was 29 h. 3D HNcoCACB data was acquired using a data matrix of 486 (t_1, ^{13}C) × 230 (t_2, ^{15}N) × 1024 (t_3, H^N) complex points and the same sweep widths as in HNCACB. The total experiment time was 30 h. 3D HNCO data was acquired using a data matrix of 136 (t_1, ^{13}C) × 300 (t_2, ^{15}N) × 1024 (t_3, H^N) complex points and sweep widths of 1057 (^{13}C),

1176 (^{15}N), and 6250 (H^N) Hz. The total experiment time was 36 h. 3D HNcaCO data was acquired using a data matrix of 148 (t_1, ^{13}C) × 300 (t_2, ^{15}N) × 1024 (t_3, H^N) complex points and sweep widths of 1359 (^{13}C), 1065 (^{15}N), and 6250 (H^N) Hz. The total experiment time was 39 h. Because the chains in SsfA(B)$_{66-97}$ are also present in SsfA(BM)$_{66-97}$, the above-mentioned 3D experiments can be used to assign SsfA(B)$_{66-97}$. However, to improve the accuracy of SsfA(B)$_{66-97}$ assignment, an additional 3D HNCACB experiment was performed using the SsfA(B)$_{66-97}$ sample. 3D HNCACB data was acquired using a data matrix of 1024 (t_1, H^N) × 230 (t_2, ^{15}N) × 486 (t_3, ^{13}C) complex points and sweep widths of 10000 (^{13}C), 1176 (^{15}N), and 6250 (H^N) Hz. The total experiment time was 96 h. A recycle delay of 0.5 s and four scans per increment were used in all 3D assignment experiments. All NMR data were processed by NMRPipe[9]. 90°-shifted sine-bell window functions were applied to all dimensions prior to zero-filling and Fourier transformation. All NMR spectra were analyzed by NMRFAM-SPARKY[10].

Sequential assignment of proline residues in SsfA(B)$_{66–97}$. The 4D HNcocancaNH (x-P-x) experiment by Wong et al.[11] correlates two backbone amide groups, i.e., $N_{i+1}H_{i+1}$, and $N_{i-1}H_{i-1}$, that are flanking a proline residue at position i. The 4D experiment was run in a 3D mode without frequency labeling in the H_{i+1} dimension. 0.26 mM ^{13}C, ^{15}N-labeled SsfA(B)$_{66-97}$ was prepared in the 20 mM sodium phosphate buffer (pH 6.0) containing 20 mM sodium chloride and 5% D_2O and placed in a 5 mm D_2O-matched Shigemi tube. The 3D hNcocancaNH data was acquired with a recycle delay of 1.5 s, forty scans per increment, a data matrix of 64 (t_1, ^{15}N) × 64 (t_2, ^{15}N) × 2048 (t_3, H^N) complex points, and sweep widths of 1176 (^{15}N), 1176 (^{15}N), and 2048 (H^N) Hz. The total experiment time was 84 h. The NMR data were processed as described in the protein backbone assignment section.

NMR analysis of synthetic peptides and FcaA(M)$_{19–26}$. ^{1}H, ^{1}H-^{1}H COSY, ^{1}H-^{1}H TOCSY, and ^{1}H-^{1}H NOESY NMR spectra of synthetic peptides and FcaA(M)$_{19-26}$ were acquired at 293.1 K. All samples were dissolved in 600 µL of d_6-DMSO at 4.0 mg/mL concentration. All data were processed and analyzed by MestReNOVA[12].

NMR experimental parameters used for synthetic peptides and FcaA(M)$_{19–26}$

**Experiment**	**Sweep width**	**Data matrix**	**Number of scans (per increment)**	**Mixing time**	**Total acquisition time**
^{1}H	17857 Hz	16384	256	-	13 m
^{1}H-^{1}H COSY	5882 Hz^	2048 (t_1, ^{1}H) × 256 (t_2, ^{1}H)	4	-	39 m
^{1}H-^{1}H TOCSY	5882 Hz^	2048 (t_1, ^{1}H) × 1024 (t_2, ^{1}H)	8	80 ms	5 h 21 m
^{1}H-^{1}H NOESY	5882 Hz^	2048 (t_1, ^{1}H) × 512 (t_2, ^{1}H)	8	400 ms	2 h 24 m

^ (in both ^{1}H dimensions)
t1 and t2 indicates direct dimension and indirect dimension, respectively.

Genome mining of pamtides. Bioinformatic analysis for RiPP-associated PIMT homologs, PAMTs, are illustrated in Figure S8 and Figure 3a. For the retrieval of PAMTs, Position-Specific Iterative BLAST (PSI-BLAST)[13] was performed in December 2021 on a non-redundant protein database in National Center for Biotechnology Information (NCBI, http://www.ncbi.nlm.nih.gov) using SsfM as a query and 1e-25 as a cut-off value for four iterations as described in Figure S8. Total 73,855 proteins were obtained, and dereplicated by the sequence identity of 70%, yielding 23,490 proteins. These proteins were aligned by MAFFT[14] 7.453 with the G-large-ins-1 algorithm and the resulting alignment was used to generate a maximum likelihood tree using FastTree 2.1.11[15]. To predict the biological role of these proteins, they were queried to Rapid ORF Description and Evaluation Online (RODEO)[16] to retrieve the information of neighbor genes. Enzymes were classified if known RiPP BGCs were found nearby; BGCs for either

lanthipeptides, lasso peptides, graspetides, or LAPs were found in the analysis. Also, genes for SurE were often found nearby, forming a *surE-pcm* gene cluster for long-term survival of cells. The tree was visualized by ETE3 3.1.2[17] with their domain architectures and their predicted roles are annotated in parallel.

Finally, 4,003 PAMTs and proteins that were dereplicated by these enzymes were further analyzed as illustrated in Figure 3a. First, neighbor genes and their sequences were retrieved using RODEO[16]. PAMTs associated in known RiPP BGCs were classified. Next, short ORFs (≤ 100 amino acids) encoded near PAMT (± 500 bps) and containing aspartate(s) were classified by their homology using a cut-off value of 1e-06 in EFI-EST (https://efi.igb.illinois.edu/efi-est/)[18]. Clusters with more than 5 peptides with conserved aspartates were assigned to the putative pamtide BGCs. Finally, proteins encoded in conserved gene clusters containing Forkhead-associated (FHA) domain-containing proteins and von Willebrand factor type A (vWA) domain-containing proteins are assigned to FHA-VWA gene clusters. Proteins were aligned by MAFFT[14] with G-large-ins-1 algorithm, a maximum-likelihood tree was built with the alignment with FastTree 2.1.11[15], and the tree was visualized by ETE3 3.1.2[17]. Precursor peptides were aligned by MAFFT[14] with E-ins-i option and sequence logos were generated using WebLogo3[19].

Protein structure prediction and analysis. Structures of full-length SsfM, OlvS, TceM, and FcaA were predicted in AlphaFold and ESMFold[20]. C-terminal domains of SsfM, OlvS, and TceM were queried to ColabFold[21]. To locate the Zn^{2+} ion in the model structure of FcaA, alphafold structure was queried in MIB (http://combio.life.nctu.edu.tw/MIB2/)[22]. Structural alignment of SsfM, OlvS, TceM with the crystal structure of PIMT from *Thermotoga maritima* (PDB 1DL5) was conducted in DALI server[23].

Metal binding assay. Stoichiometry between binding of zinc ion and peptides/proteins were determined using 4-(2-pyridylazo)resorcinol (PAR)[24] as follows: Zn^{2+}-2 PAR complexes were first prepared by mixing 20 μM of $ZnCl_2$, 200 μM of PAR in 20 mM Tris-HCl pH 8.0 and 100 mM NaCl to a final volume of 50 μL. To dissociate the complex, the mixture was 2-fold diluted by adding 50 μL of FcaA, FcaA(M), IAA-labeled FcaA, FcaM, or EDTA. Final concentrations of the added peptides/proteins/EDTA were 1–15 μM. Changes in absorbance at 500 nm was observed using Infinite M200 pro (Tecan, Switzerland).

Supplementary Figures

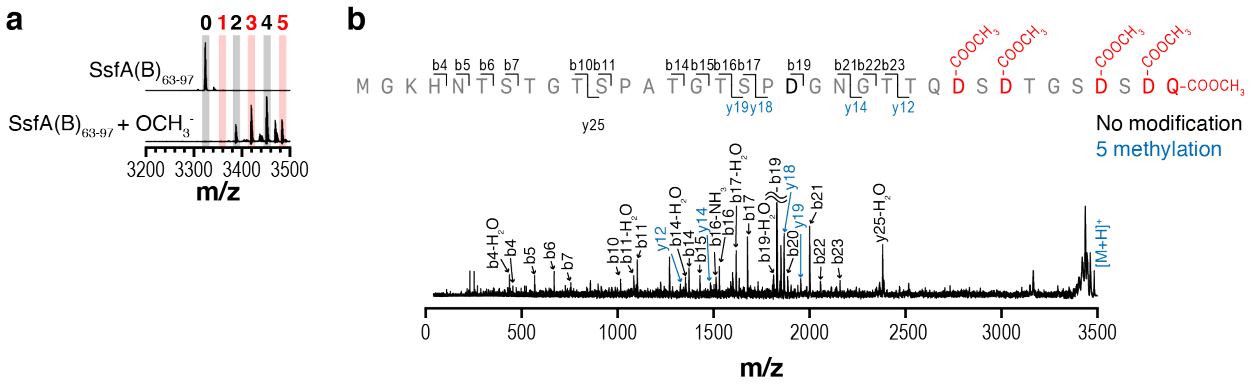

Figure S1. Mass analysis of the methanolyzed SsfA(B)$_{63–97}$. (**a**) MALDI-TOF-MS spectra of the GluC-digested SsfA(B) (top) and its methanolized products (bottom). The numbers of added methanol molecules are specified above each peak. (**b**) MALDI-TOF/TOF-MS spectrum of five-fold methanolized SsfA(B)$_{63–97}$. Observed ions are colored according to the numbers of added methyl groups in each fragment (black, 0; blue, 5). Calculated and observed mass values can be found in Supplementary Dataset 1.

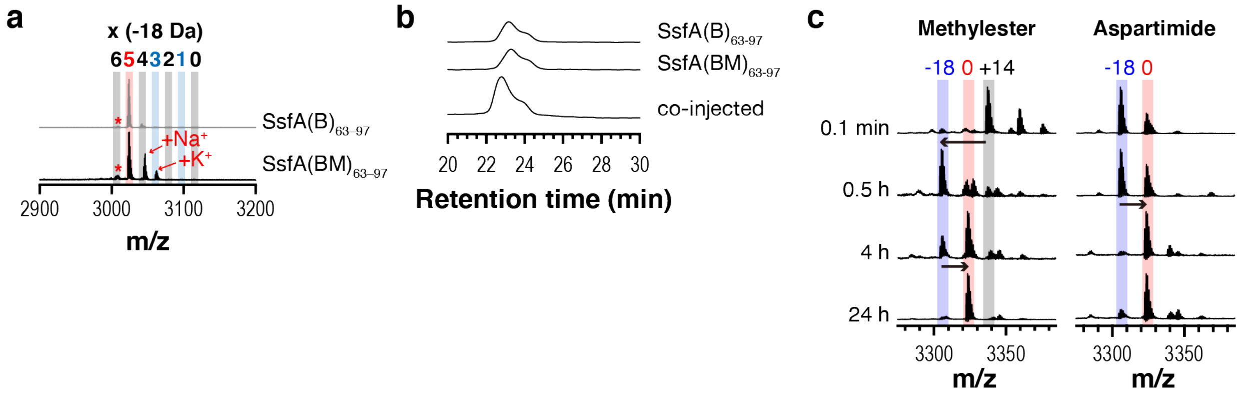

Figure S2. MS and LC analysis of SsfA(B) and SsfA(BM) variants. (**a**) MALDI-TOF-MS spectra of GluC-digested SsfA(BM) (bottom). Mass spectrum of SsfA(B)$_{63–97}$ in Figure S1a is shown above (grey line) for comparison. Numbers of eliminated water molecules are designated above spectra. Red asterisks indicate the mass peaks arising from the laser-induced deamination, which are not relevant to the activity of SsfB or SsfM. (**b**) HPLC analysis of the GluC-digested SsfA(B) and SsfA(BM). (**c**) MALDI-TOF-MS spectra of the aspartyl *O*-methylester-containing SsfA(BM)$_{63–97}$ (methylester, left) and aspartimide-containing SsfA(BM)$_{63–97}$ (aspartimide, right). Intermediates were dissolved in 10 mM Tris-HCl pH 8.0 to a final concentration of 10–50 µM at 25 °C. Solutions were desalted and molecular weights were monitored by mass analyzer at each time point. Calculated and observed mass values can be found in Supplementary Dataset 1.

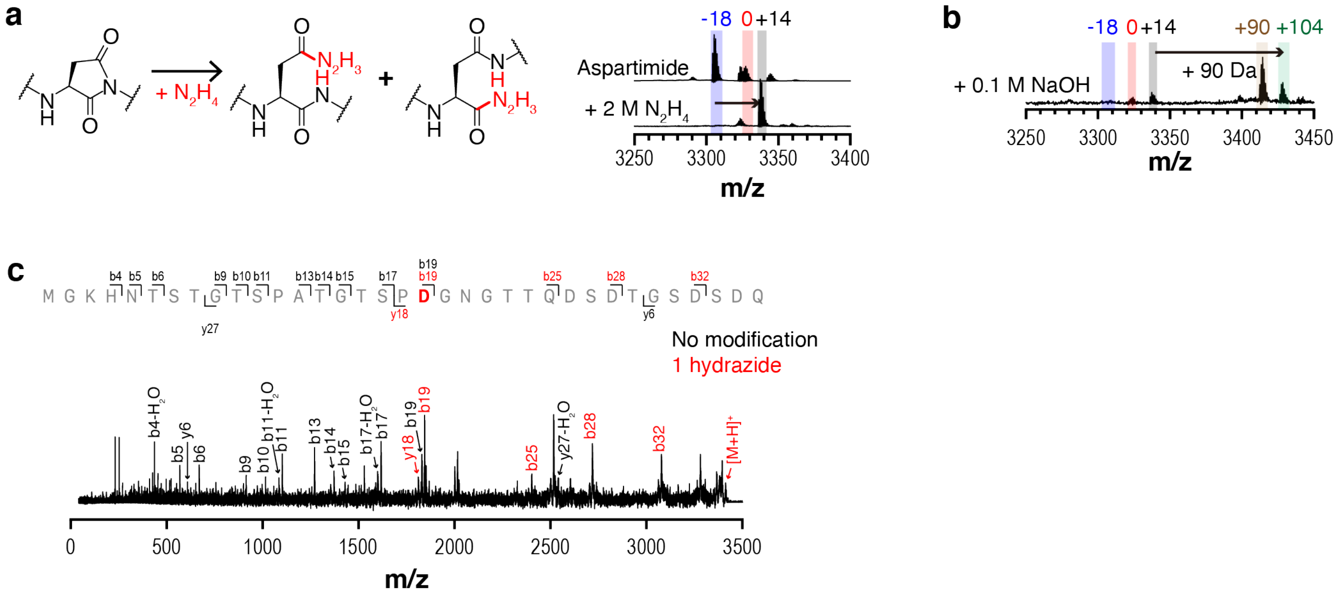

Figure S3. Mass analysis of hydrazide-containing SsfA(BM)$_{63–97}$. (**a**) Reaction scheme of the hydrazine addition to the aspartimide (left) and MALDI-TOF-MS spectrum of the hydrazide-containing peptide (right). Relative mass changes are annotated above peaks. (**b-c**) MALDI-TOF-MS spectrum (**b**) and MALDI-TOF/TOF-MS spectrum (**c**) were obtained to locate the hydrazide. Peptide was dissolved in 0.1 M NaOH solution and incubated at 25 °C for 30 minutes. Relative mass value changes to SsfA(BM)$_{63–97}$ are given above each peak. Ions are colored according to the numbers of hydrazide group in the fragments (0, black; 1, red). Observed and calculated mass values can be found in Supplementary Dataset 1.

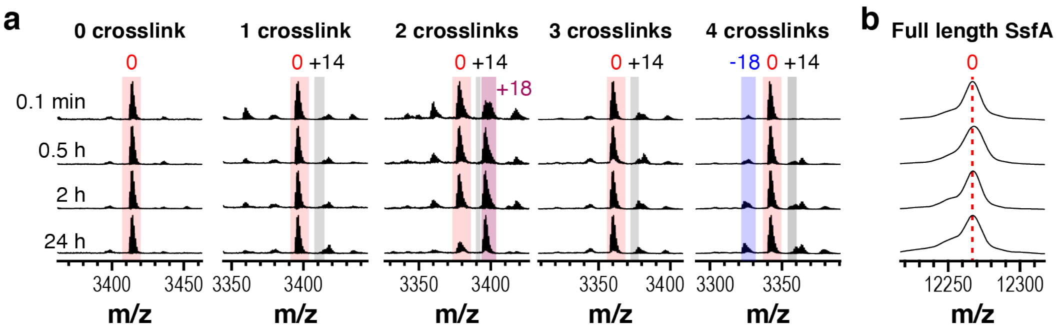

Figure S4. MALDI-TOF-MS spectra of SsfM-mediated modification reaction of SsfA variants. Either one of partially crosslinked core peptides (**a**) or intact SsfA (**b**) was incubated with SsfM at a same condition as in Figure 2c. Relative mass value changes to the unreacted substrates are given above each peak. Observed and calculated mass values can be found in Supplementary Dataset 1.

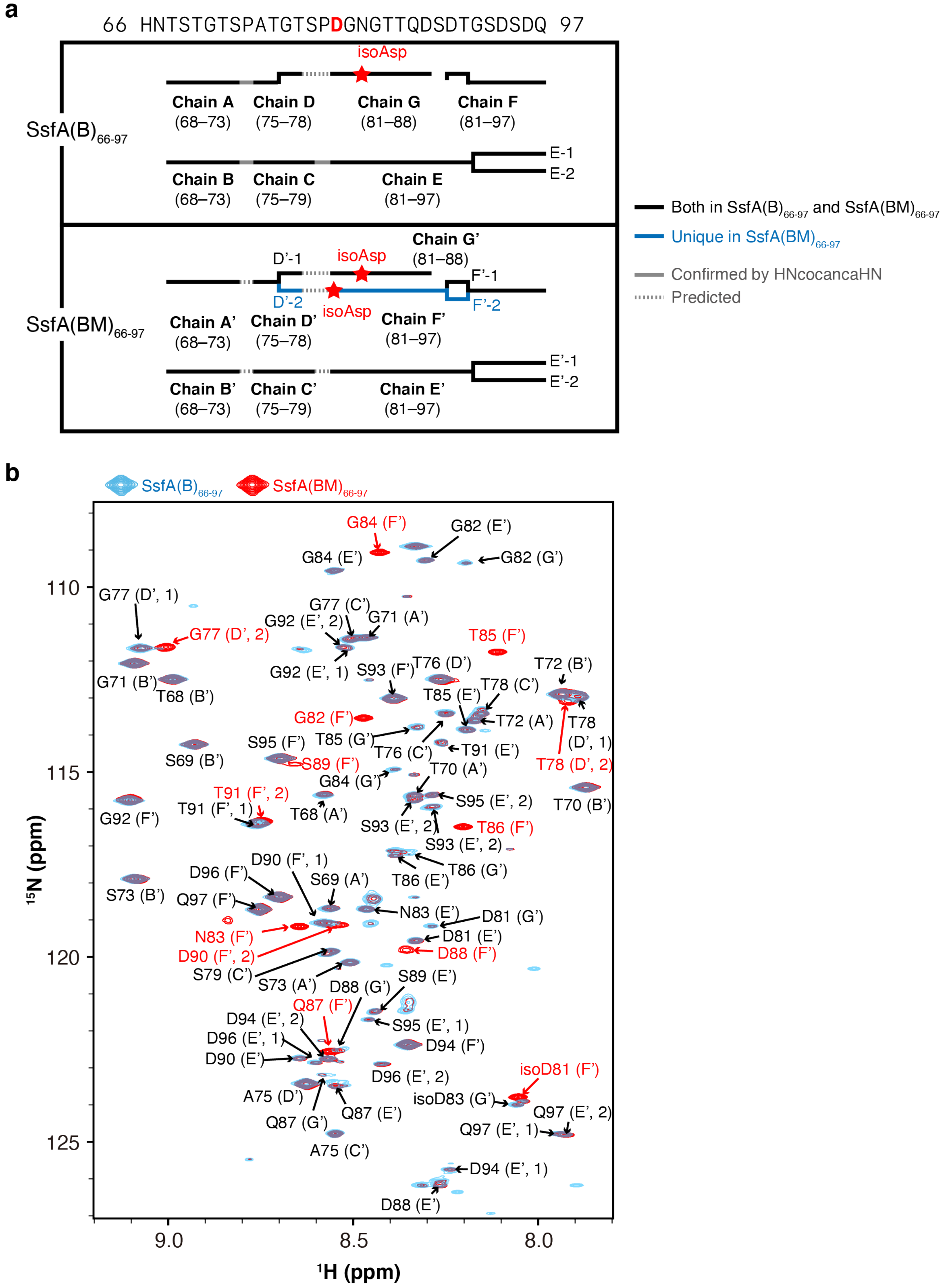

Figure S5. Assigned Chains and ^{1}H-^{15}N HSQC spectra of SsfA(B)$_{66\text{–}97}$ and SsfA(BM)$_{66\text{–}97}$. (**a**) Summary of the NMR-assigned chains. Signals that are observed in both SsfA(B)$_{66\text{–}97}$ and SsfA(BM)$_{66\text{–}97}$ spectra are annotated in black, while those exclusively observed in the spectrum of SsfA(BM)$_{66\text{–}97}$ are colored in blue. Seven independent peptide fragments were observed (chain A, B, A', and B', Thr68–Ser73; chain C and C', Ala75–Ser79; chain D and D', Ala75–Thr78; chain E, F, E', and F', Asp81–Gln97 or its smaller

part; chain G and G’, Asp81–Asp88). Multipe NMR resonances originated from a single residue, suggesting the presence of conformational isomers or different chemical structures. Observed isoaspartates are annotated (See Figure 2d, Figure S6-S7 for the detail). Gray solid lines indicate the connectivity confirmed by hNcocancaNH (See Methods for the detail) and gray dashed lines denote the predicted connectivity between peptide chains. (**b**) Overlaid ^{1}H-^{15}N HSQC spectra of SsfA(B)$_{66–97}$ (blue) and SsfA(BM)$_{66–97}$ (red). Signals from the spectrum of SsfA(BM)$_{66–97}$ are labeled, with color indicating its presence in the spectrum of SsfA(B)$_{66–97}$ (black, observed in both spectra; red, exclusively present in SsfA(BM)$_{66–97}$). Chemical shift values can be found in Supplementary Dataset 1.

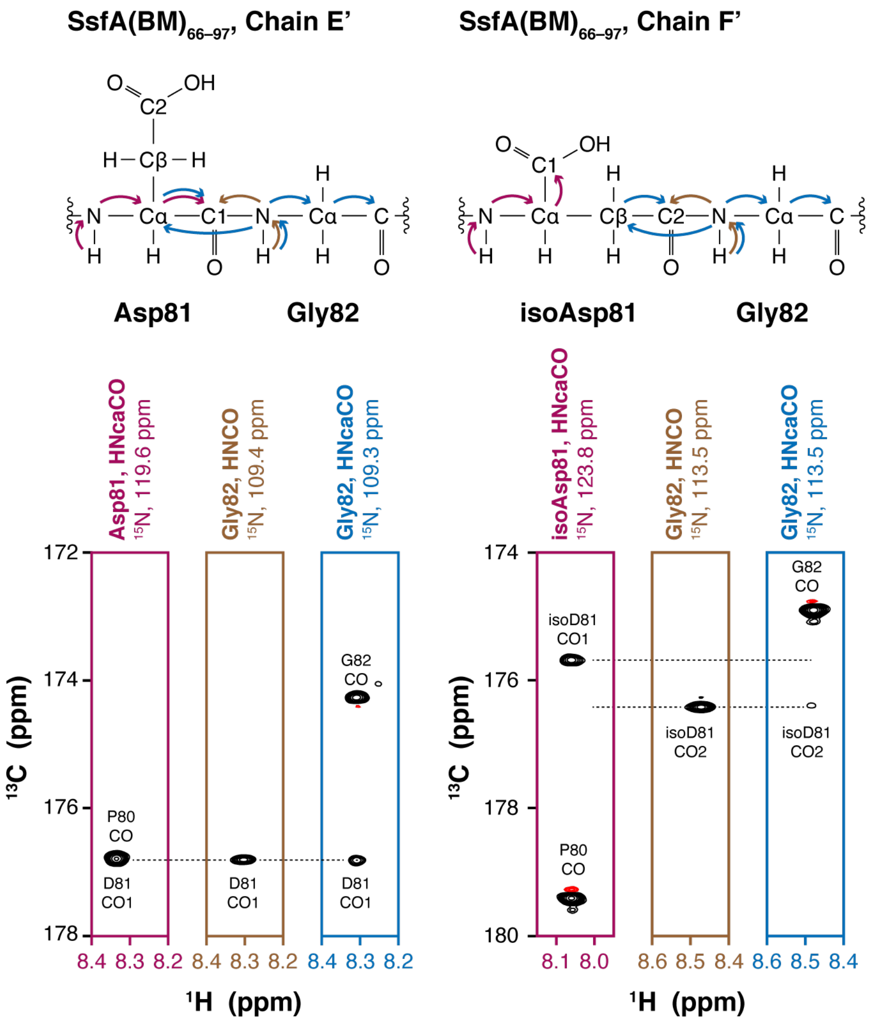

Figure S6. HNcaCO and HNCO analysis of SsfA(BM)$_{66–97}$ chain E'/F'. Magnetization transfer in Asp-Gly and isoAsp-Gly for HNcaCO/HNCO experiments (top) and HNcaCO/HNCO strip plots indicating the presence of aspartate and isoaspartate (bottom). Chemical shift values can be found in Supplementary Dataset 1.

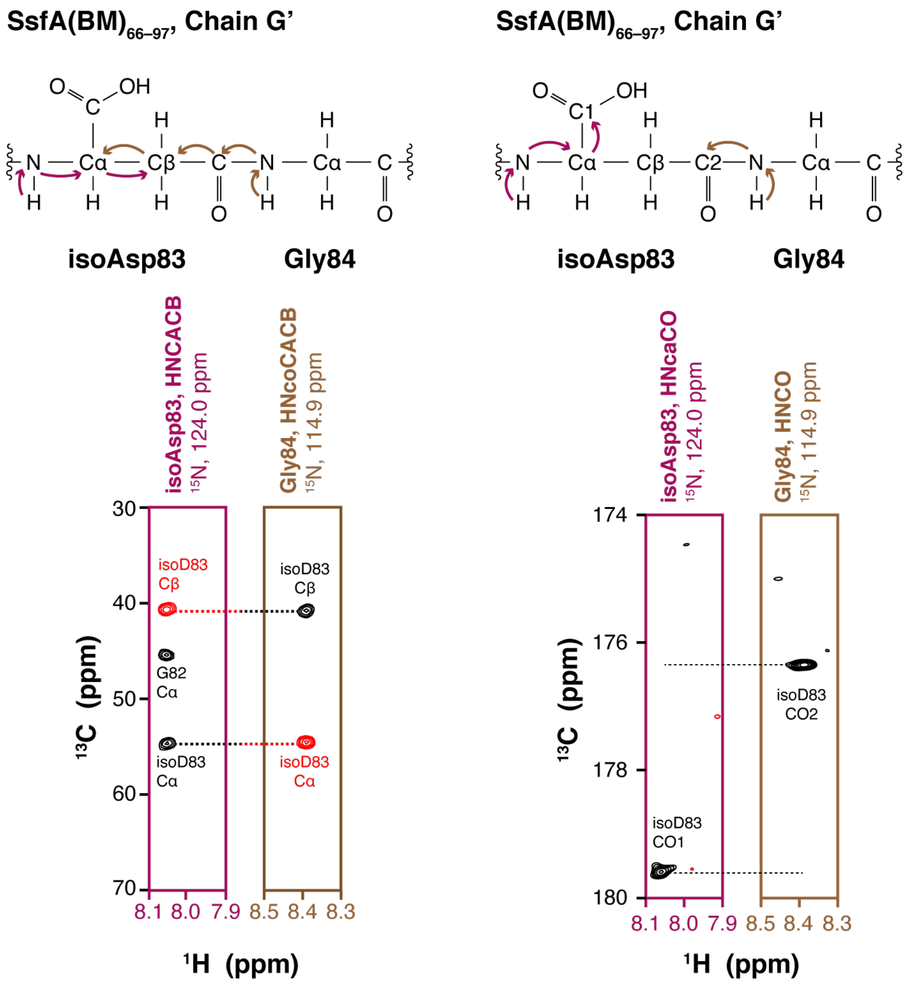

Figure S7. HNCACB, HNcoCACB, HNCO, and HNcaCO analysis of SsfA(BM)$_{66–97}$ chain G'. Magnetization transfer in isoAsp-Gly for HNCACB, HNcoCACB, HNcaCO, and HNCO experiments (top) and strip plots taken from the $^{1}H^{N}$-^{13}C planes indicating the presence of isoaspartate (bottom). Chemical shift values can be found in Supplementary Dataset 1.

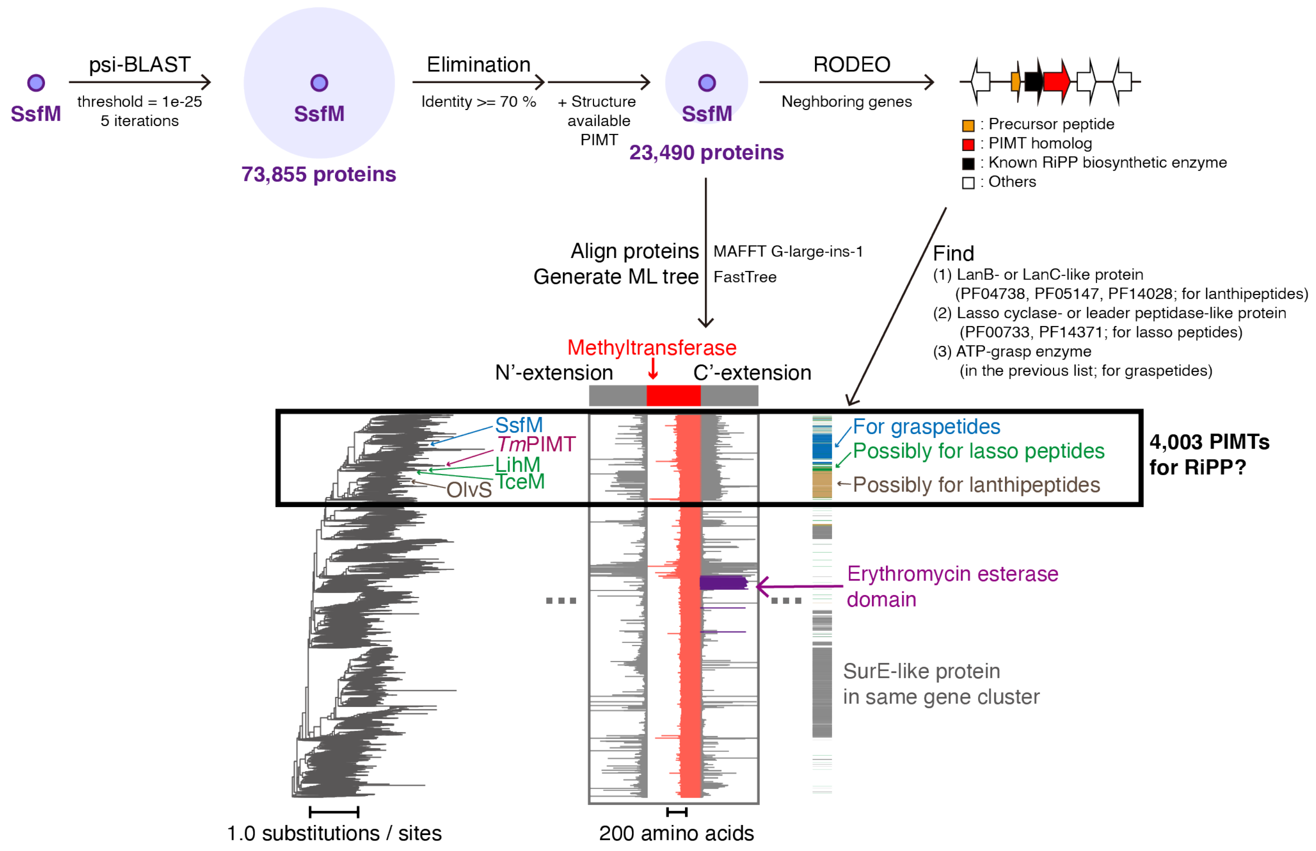

Figure S8. Bioinformatic analysis workflow of PIMTs and RiPP-associated PIMT homologs. SsfM homologs were aligned and visualized in a maximum likelihood tree. Protein architectures and putative biological pathway of proteins are illustrated together. See **Experimental Procedures** section for the details.

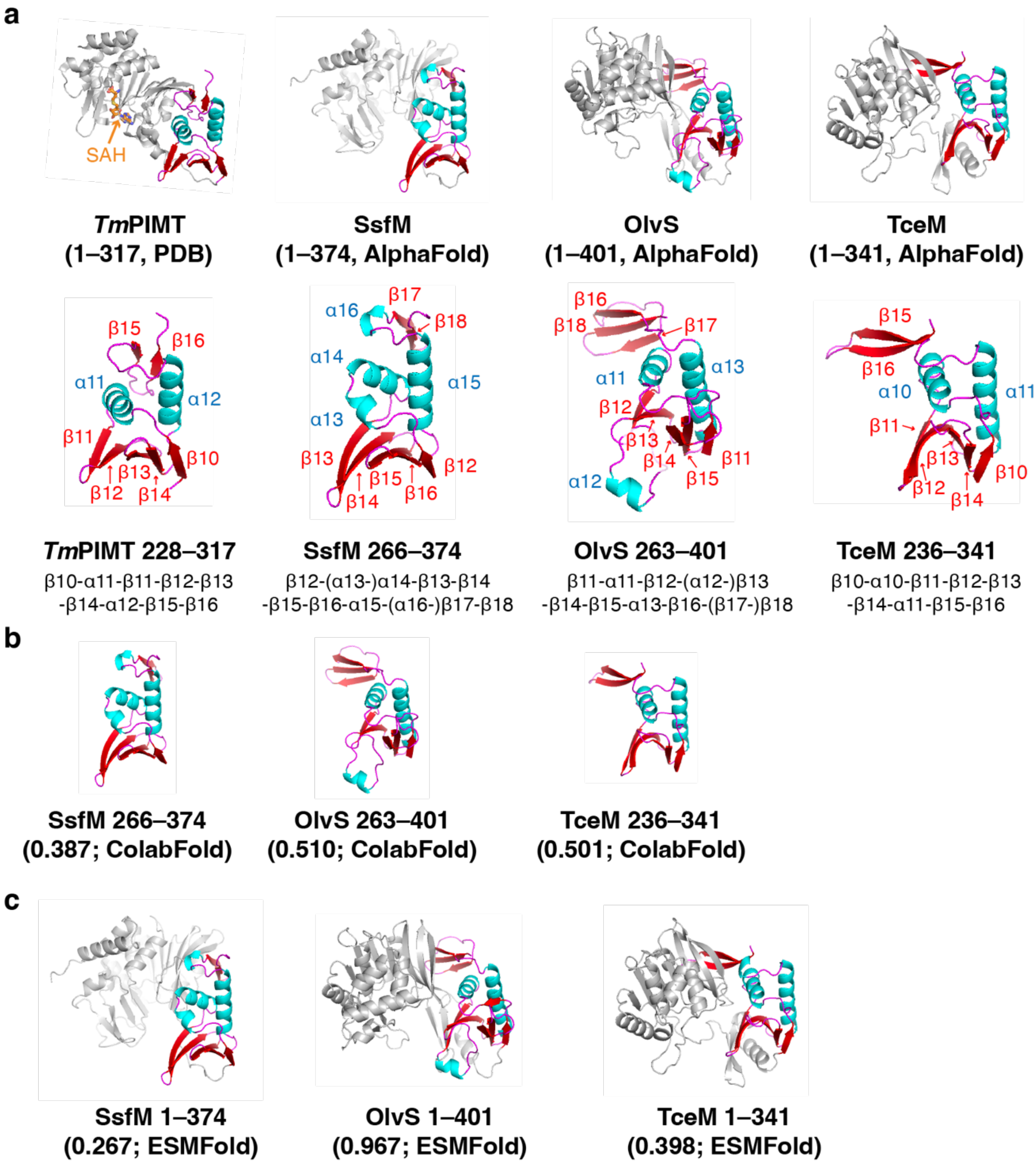

d

	*Tm*PIMT	SsfM	OlvS	TceM
*Tm*PIMT				
SsfM	4.5 (2.8)			
OlvS	3.6 (3.7)	4.8 (3.5)		
TceM	5.7 (3.1)	3.4 (4.4)	7.3 (3.4)	

Z-score (RMSD, Å)

Figure S9. Structures of the C-terminal domains in model RiPP-associated PIMT homologs. (**a**) Crystal structure of PIMT from *Thermotoga maritima* (PDB 1DL5)[25] and AlphaFold structures of full-length SsfM, OlvS, and TceM. C-terminal domains in the proteins are shown below. Conserved secondary structures are designated, while those in parentheses are uniquely observed in each protein. (**b**) ColabFold structures generated by querying sequences of the C-terminal domains of SsfM, OlvS, and TceM. Values

in parentheses are root mean square deviation (RMSD) values to the corresponding structures in A (units, Å). (**c**) ESMFold structures of full-length SsfM, OlvS, and TceM. RMSD values of the colored C-terminal domain to those in the bottom of A are given in parentheses (units, Å). (**d**) Z-score and RMSD values of the C-terminal domains illustrated in **a**. C-terminal domains were annotated by color (gray, methyltransferase; cyan, α helix; red, β sheet; magenta, loop).

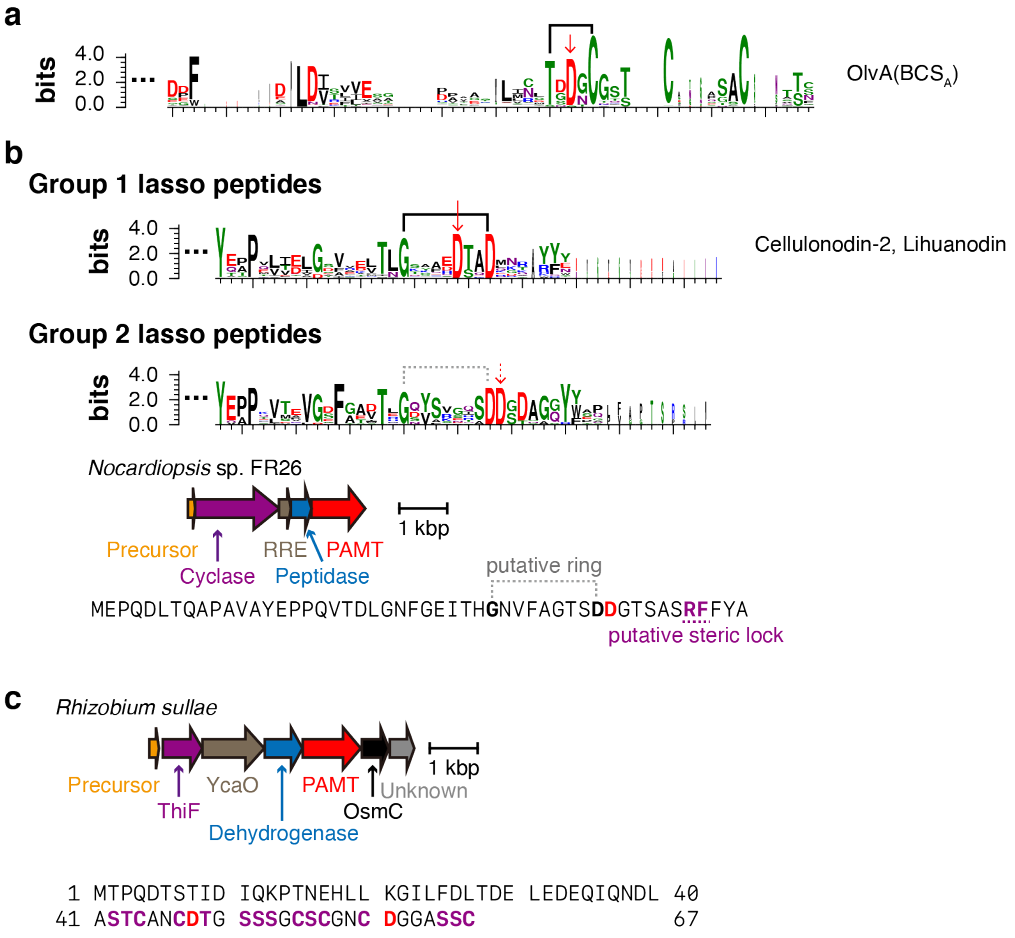

Figure S10. Features of PAMT-associated RiPPs. (**a–b**) Sequence logos of precursors for lanthipeptides (**a**) and lasso peptides (**b**). Previously identified thioether or isopeptide bond in lanthipeptides or group 1 lasso peptides are shown in black brackets, respectively. Predicted isopeptide bond in group 2 lasso peptides are drawn in gray dashed bracket. Modified aspartates in mature peptides are highlighted in red solid arrows, while predicted one in group 2 lasso peptide is highlighted in red dashed arrows. Biochemically characterized members of lanthipeptides and group 1 lasso peptides are annotated.[26] A model BGC and precursor for the group 2 lasso peptides are shown below. (**c**) A Biosynthetic gene cluster of a LAP containing a PAMT-encoding gene. Arrows are color-coded according to the conserved protein domain. Precursor peptide sequences are given below each BGCs. Putative modified aspartates in each precursor are highlighted in red.

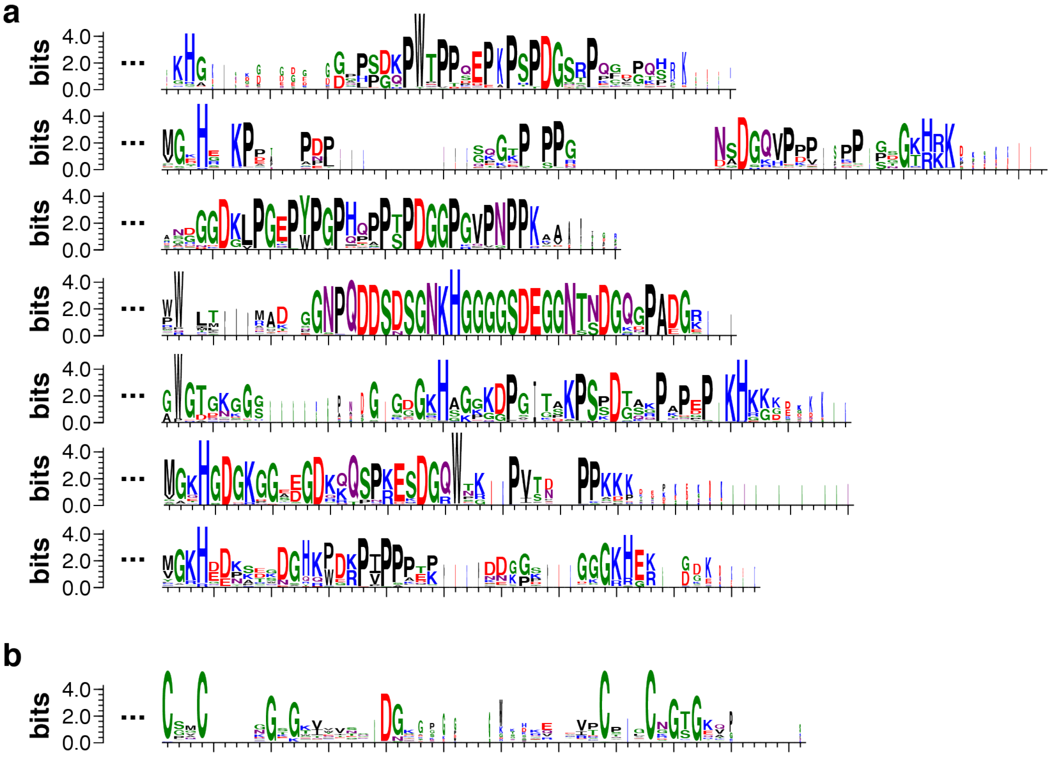

Figure S11. Sequence logos of precursor peptides for pamtides. Logos of type I precursors for seven subgroups whose members exceed 50 (**a**) and type II precursors (**b**) were generated by WebLogo.[19]

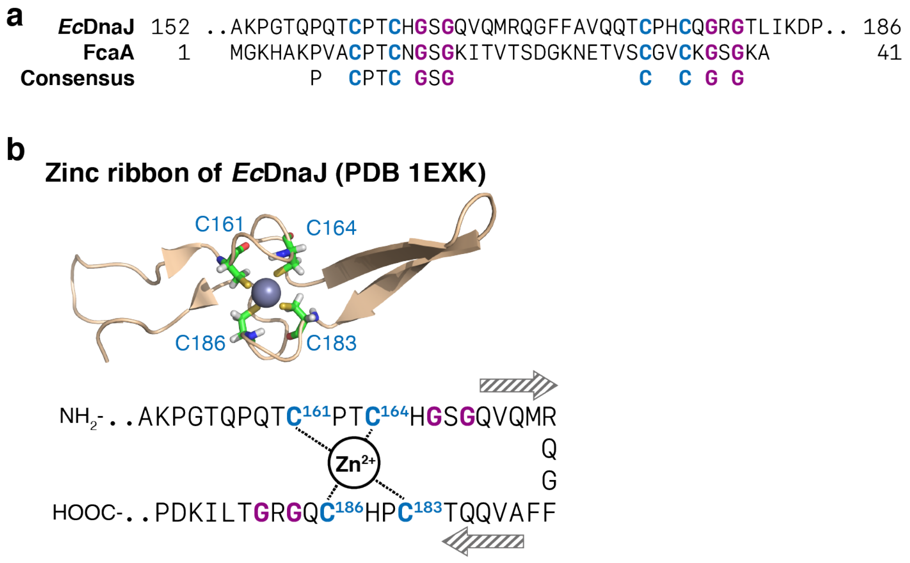

Figure S12. Structure of zinc ribbon. (**a**) Sequence alignment of zinc ribbon from *E. coli* DnaJ (*Ec*DnaJ) and FcaA. Commonly observed residues are shown below, and conserved CxxCxGxG motifs are highlighted (C, blue; G, purple). (**b**) Structure of the zinc ribbon of *Ec*DNAJ (PDB 1EXK).[27] Observed secondary structural elements and metal-ligand interactions are shown at the right.

a

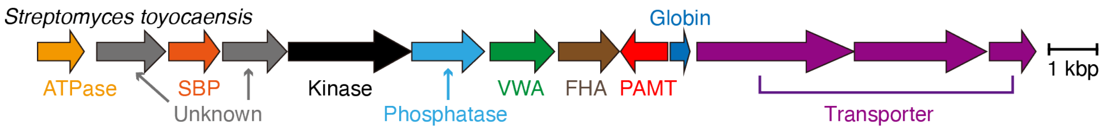

FHA

```
001 MPTCPNGHQSGSDDWCEVCGHRMAGAVPPPPPPPPAPGGGYGFPPPGGPGGPGGPGGYGGPGGYGGPGGPGGPGGPGGRT 080
081 ELCPQCRTPREGGAPFCEECRWNFLTNTATSYTPAAPRPPAPGPGPGGPFQQQPPGPSYGGDSFDYQGSRPSQMNRPAEP 160
161 IPPGPPGFGGDPSRPVPPPPGPGGHGGPGGHGGHGGPGAPQAFQPGPPAPPGFPQDTGRPQQGGPSFGGGDDDWVISPPG 240
241 GPGGPGGPGGPNGPGQGGGYGYPQPGGTQAPPGPGGFPQQPRPPQGPATWTATIGPDREYFMAMMQRSGPEAAGLNLPAY 320
321 SPEQQRQLTGNQITIGRRRHSTGDTPDIDLSTPPEDPGVSHQHAVLVQQPDGTWAVVDQNSTNGTTVNGSDEPIQPFVPV 400
401 PLQDGDRVHVGAWTTITIRRG 421
```

b

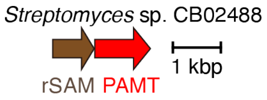

Figure S13. Conserved PAMT-associated gene clusters. Representative gene clusters of FHA/VWA-associated PAMT (**a**) and rSAM-associated PAMT (**b**) are shown. Arrows are color-coded according to the predicted protein domain. Sequence of an FHA domain-containing protein is shown below with colors illustrating the P/G-rich region (blue) and aspartates in the region (red).

Figure S14. NMR spectra of FcaA(M)19–26. FcaA(M)19–26 was analyzed by ^{1}H (**a**), ^{1}H-^{1}H COSY (**b**), ^{1}H-^{1}H TOCSY (**c**), and ^{1}H-^{1}H NOESY (**d**). Chemical shift values can be found in Supplementary Dataset 1.

a

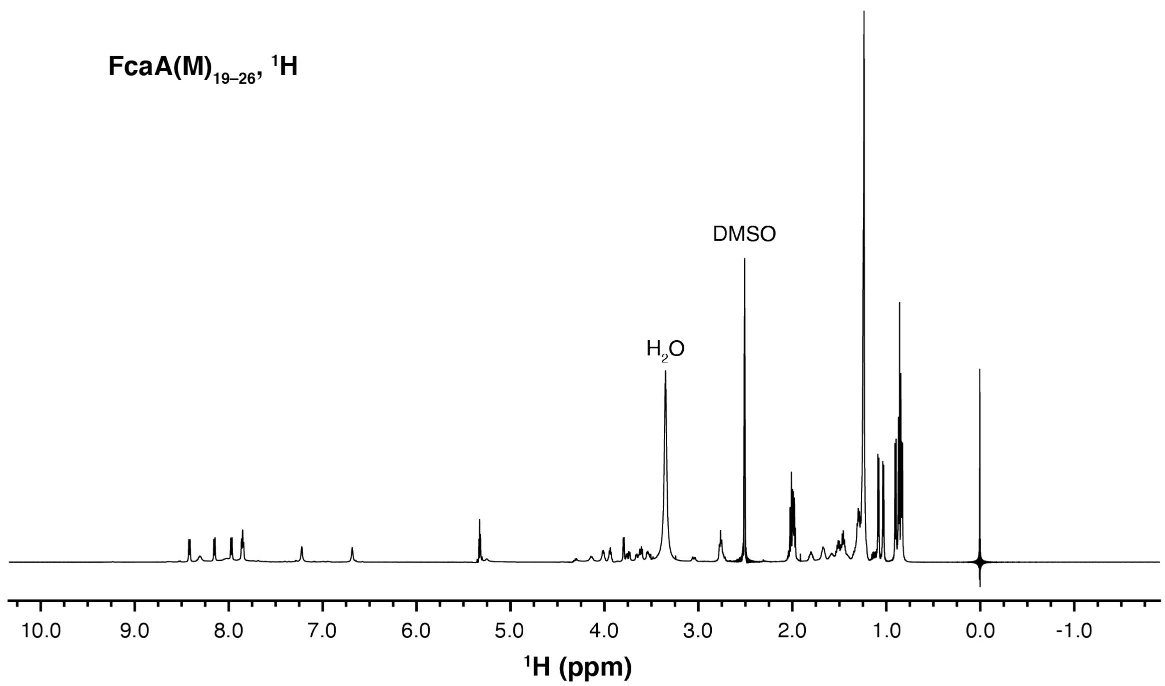

b

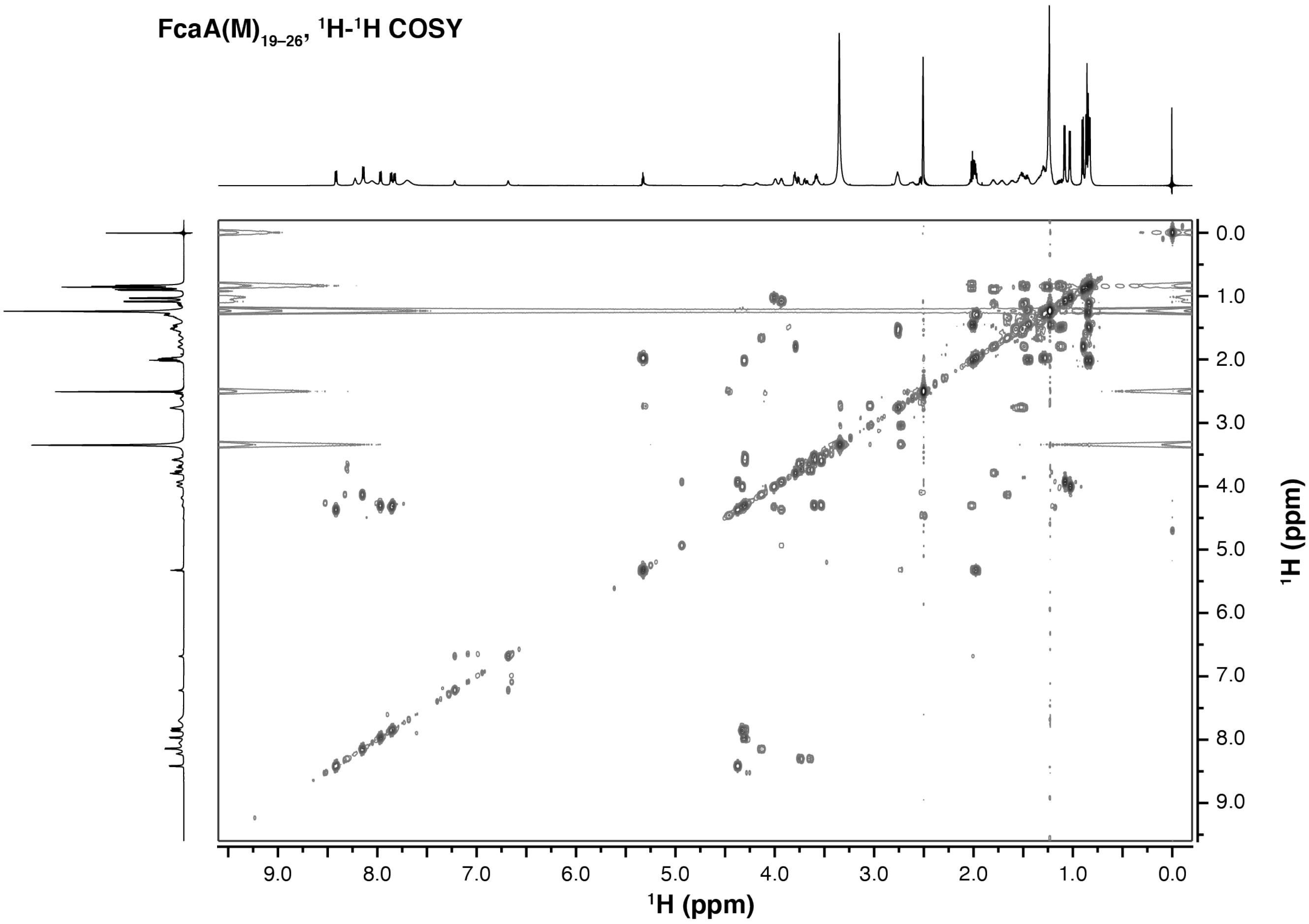

c

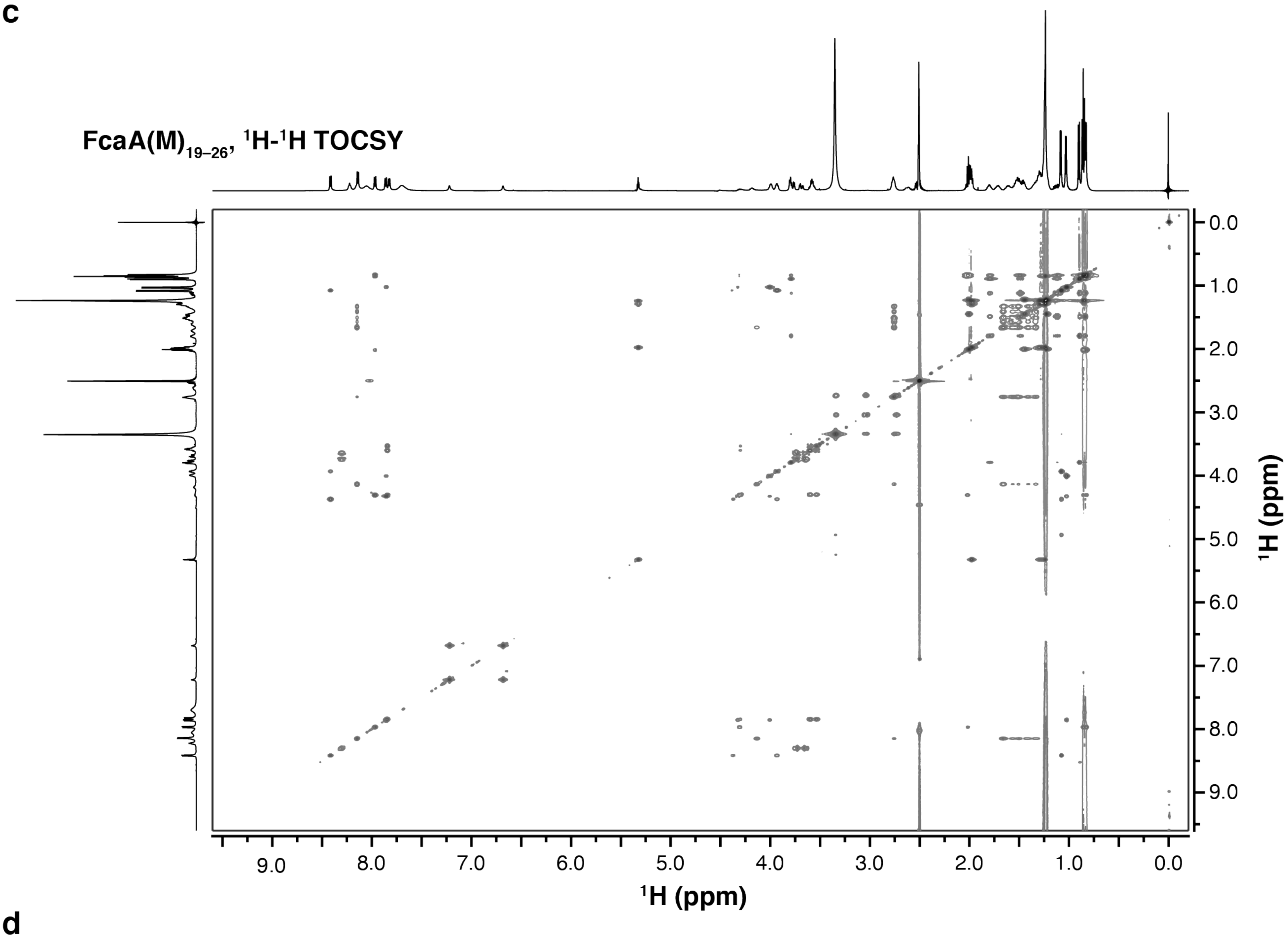
FcaA(M)$_{19–26}$, ^{1}H-^{1}H TOCSY
9.0
8.0
7.0
6.0
5.0
4.0
3.0
2.0
1.0
0.0
^{1}H (ppm)
^{1}H (ppm)

d

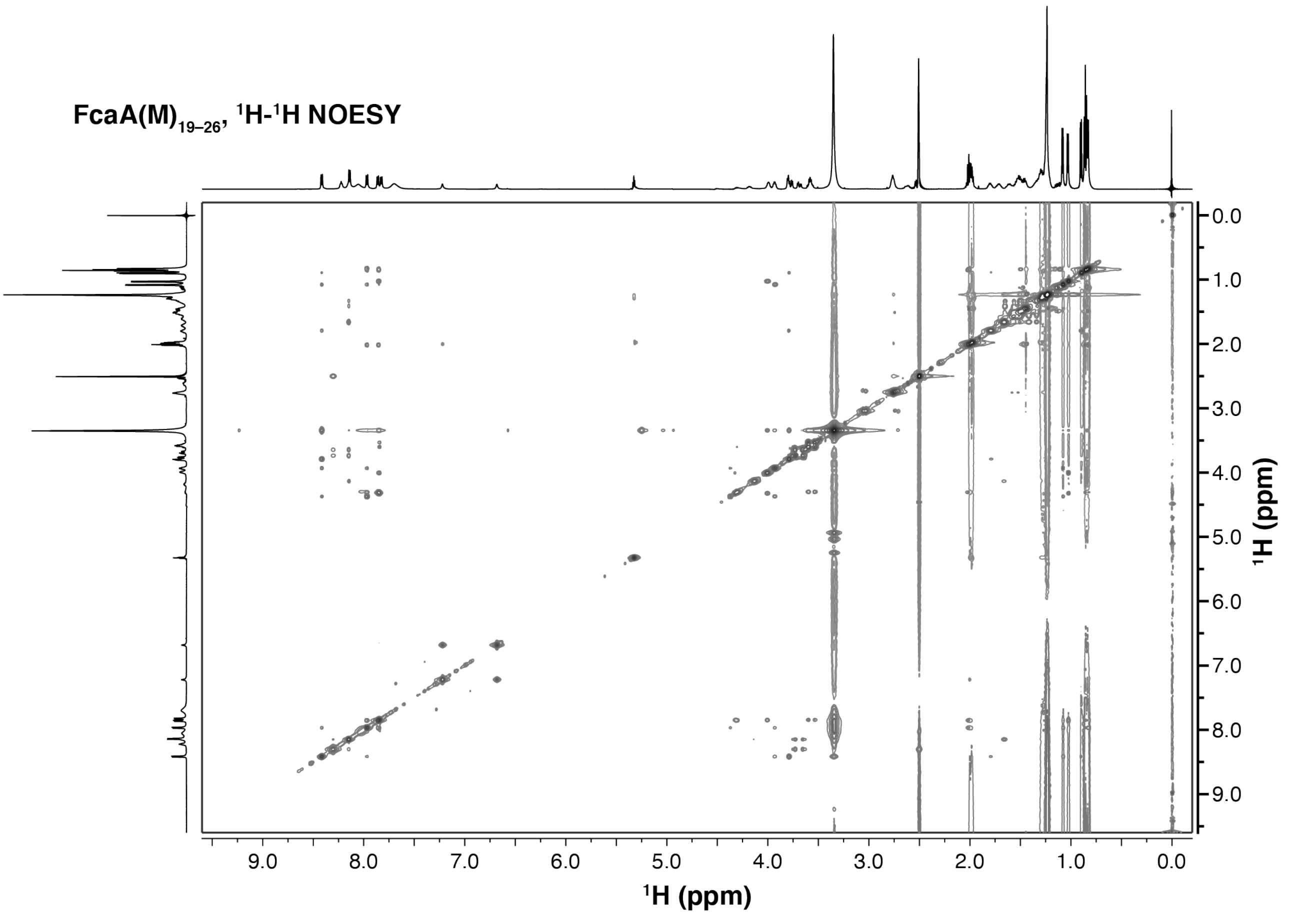
FcaA(M)$_{19–26}$, ^{1}H-^{1}H NOESY
9.0
8.0
7.0
6.0
5.0
4.0
3.0
2.0
1.0
0.0
^{1}H (ppm)
^{1}H (ppm)

Figure S15. NMR analysis of synthetic ITVTSDGK and ITVTS(isoD)GK. (**a–b**)TOCSY and NOESY spectra of ITVTSDGK (**a**) and ITVTS(isoD)GK (**b**). In ITVTSDGK, of the two possible NOE signals, one between D6 H^{α} and G7 H^{N} (red solid circle, **a**) and the other between D6 H^{β} and G7 H^{N} (black dotted circle, **a**), the former signal was uniquely observed. On the other hand, an NOE signal was observed between isoD6 H^{β} and G7 H^{N} (red solid circle, **b**), instead of isoD6 H^{α} and G7 H^{N} (black dotted circle, **b**). (**c-j**) Raw ^{1}H (**c**, ITVTSDGK; **g**, ITVTS(isoD)GK), ^{1}H-^{1}H COSY (**d**, ITVTSDGK; **h**, ITVTS(isoD)GK), ^{1}H-^{1}H TOCSY (**e**, ITVTSDGK; **i**, ITVTS(isoD)GK), and ^{1}H-^{1}H NOESY (**f**, ITVTSDGK; **j**, ITVTS(isoD)GK) spectra. Observed chemical shift values can be found in Supplementary Dataset 1.

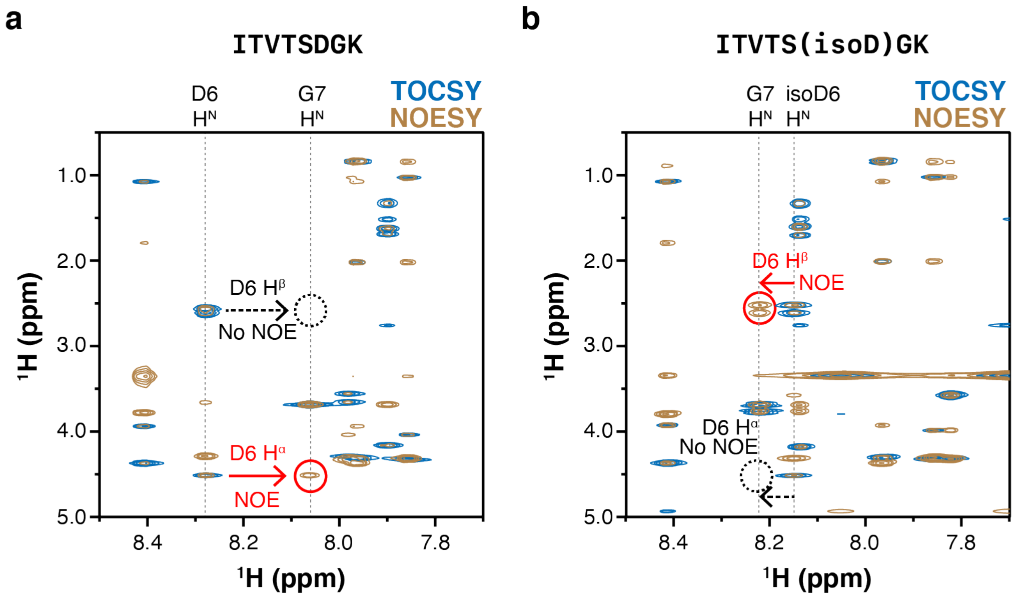

c

ITVTSDGK, ¹H

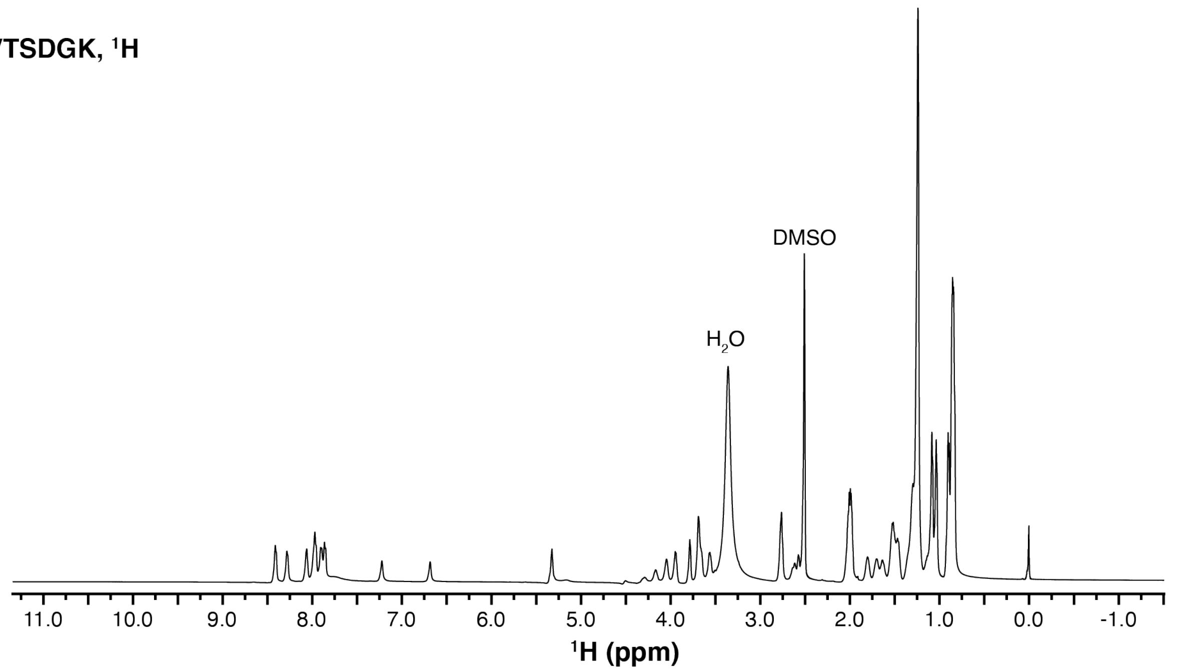

d

ITVTSDGK, ¹H-¹H COSY

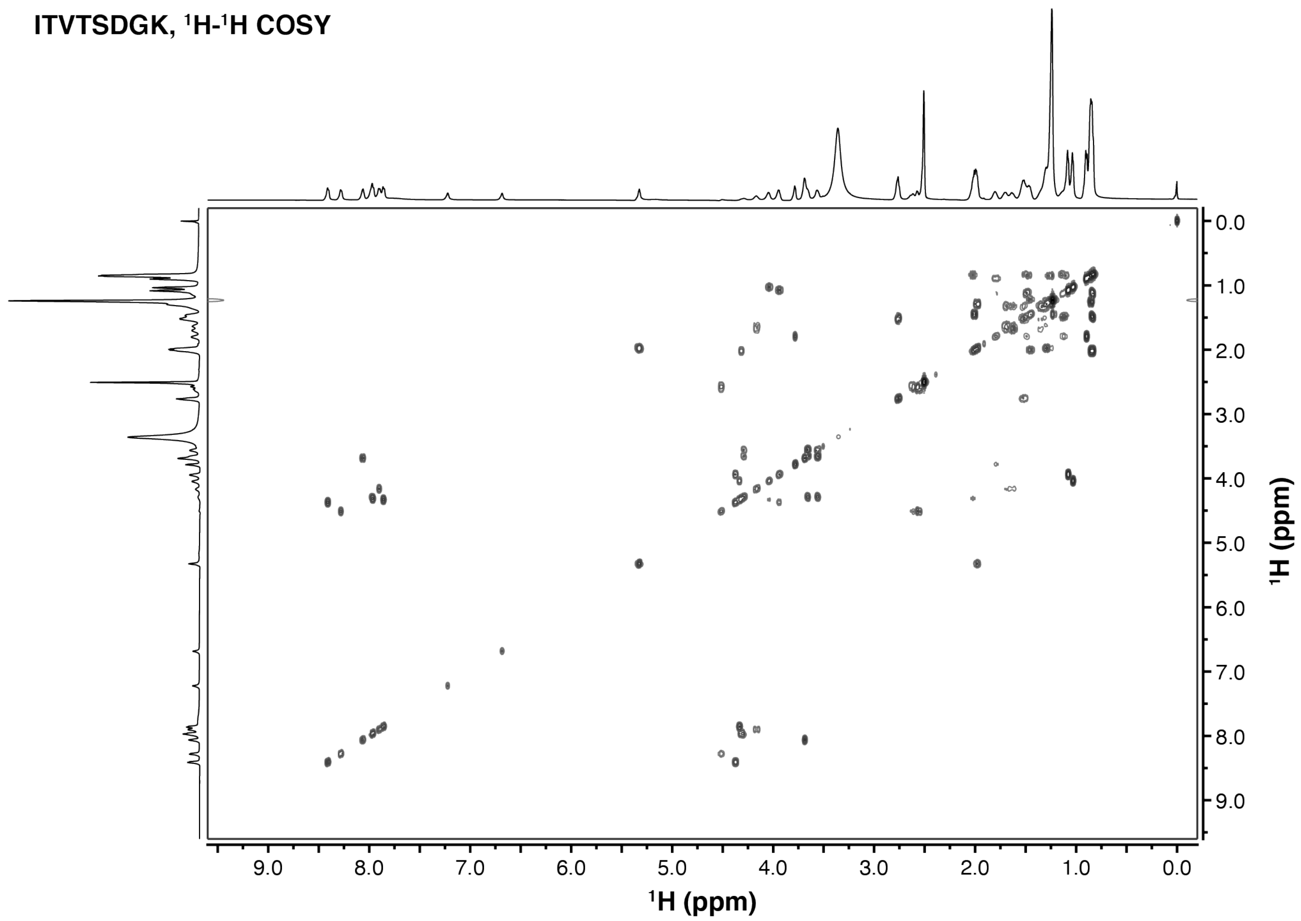

e

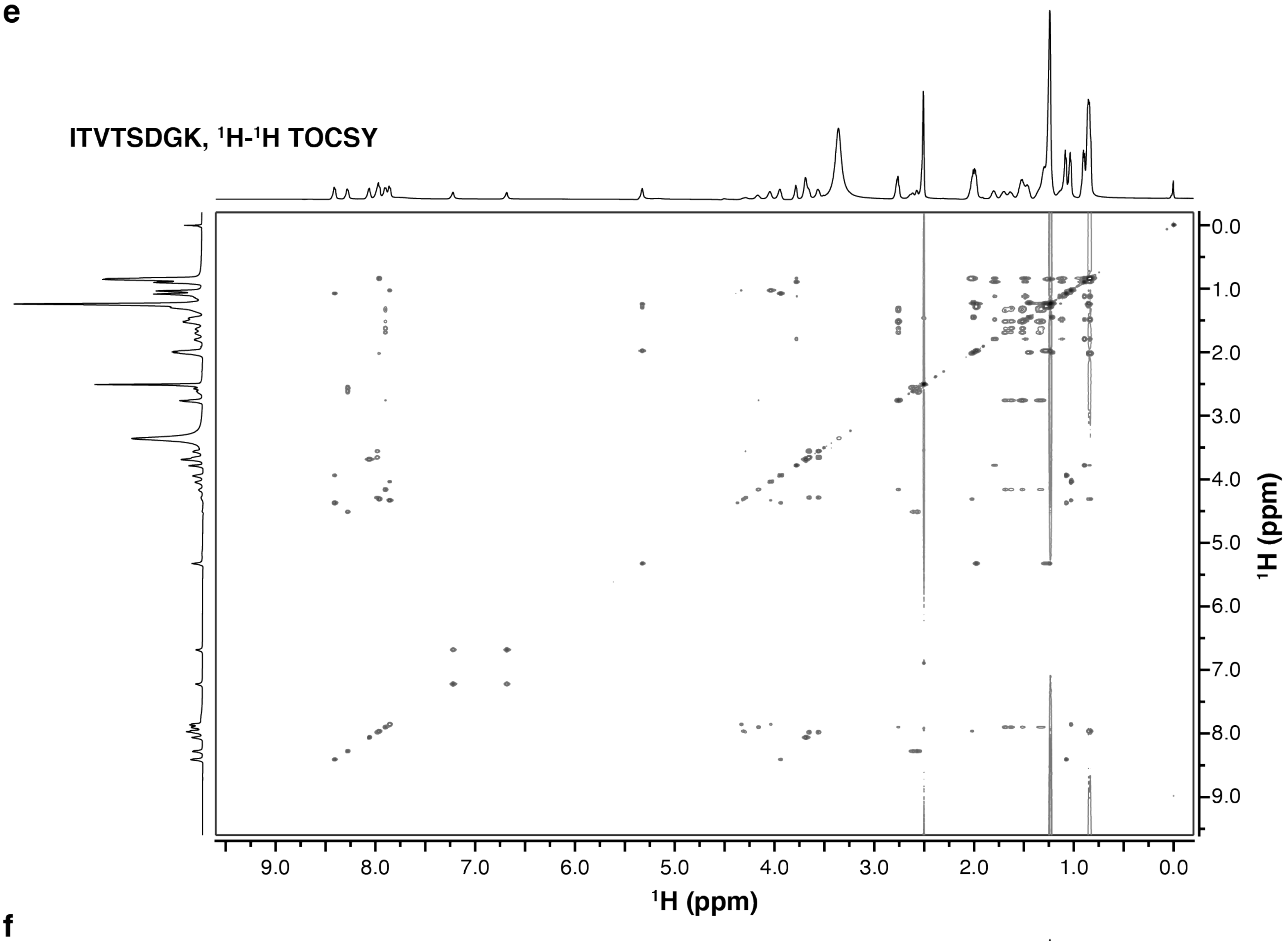
ITVTSDGK, ¹H-¹H TOCSY
¹H (ppm)
¹H (ppm)

f

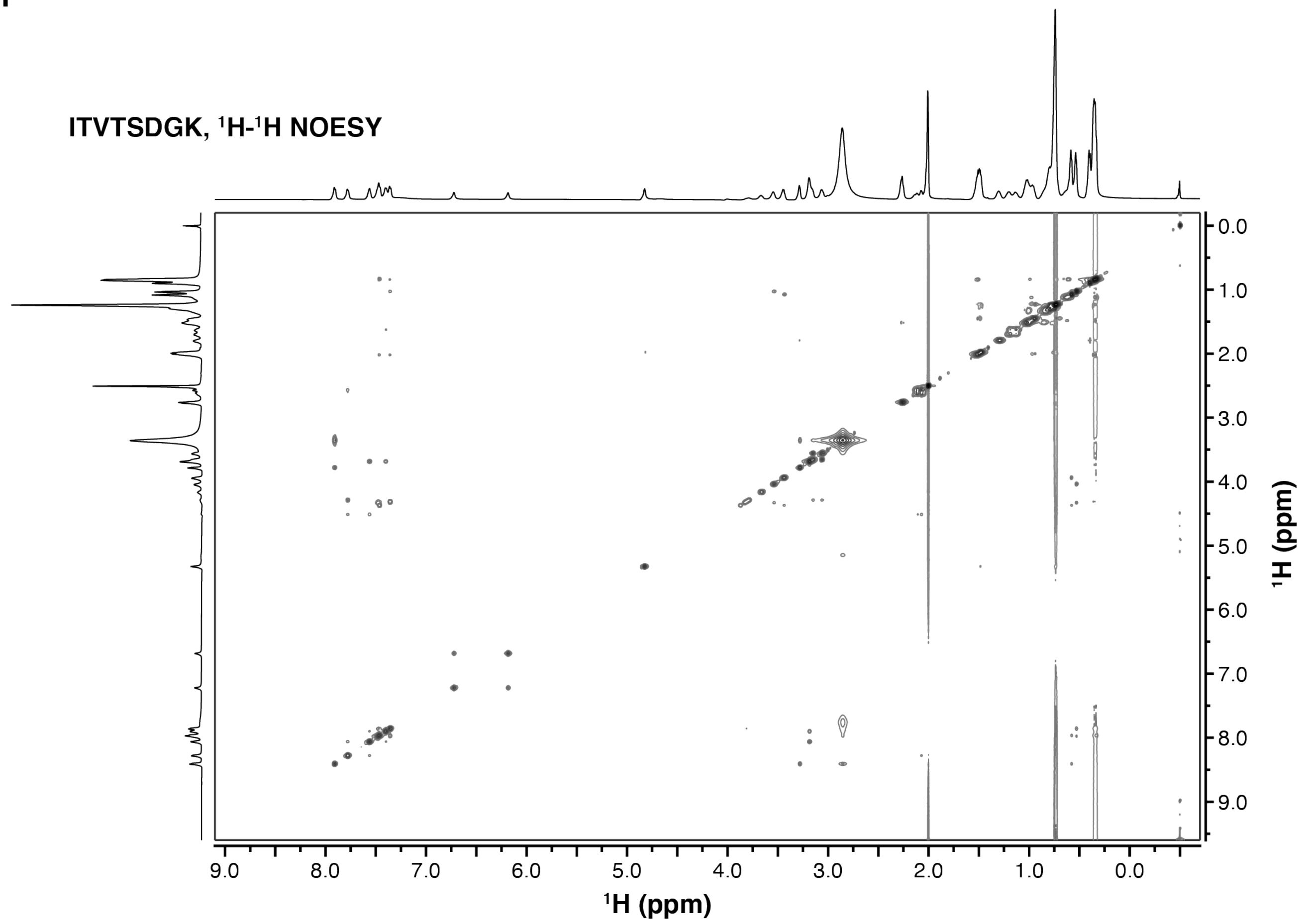
ITVTSDGK, ¹H-¹H NOESY
¹H (ppm)
¹H (ppm)

g

ITVTS(isoD)GK, ¹H

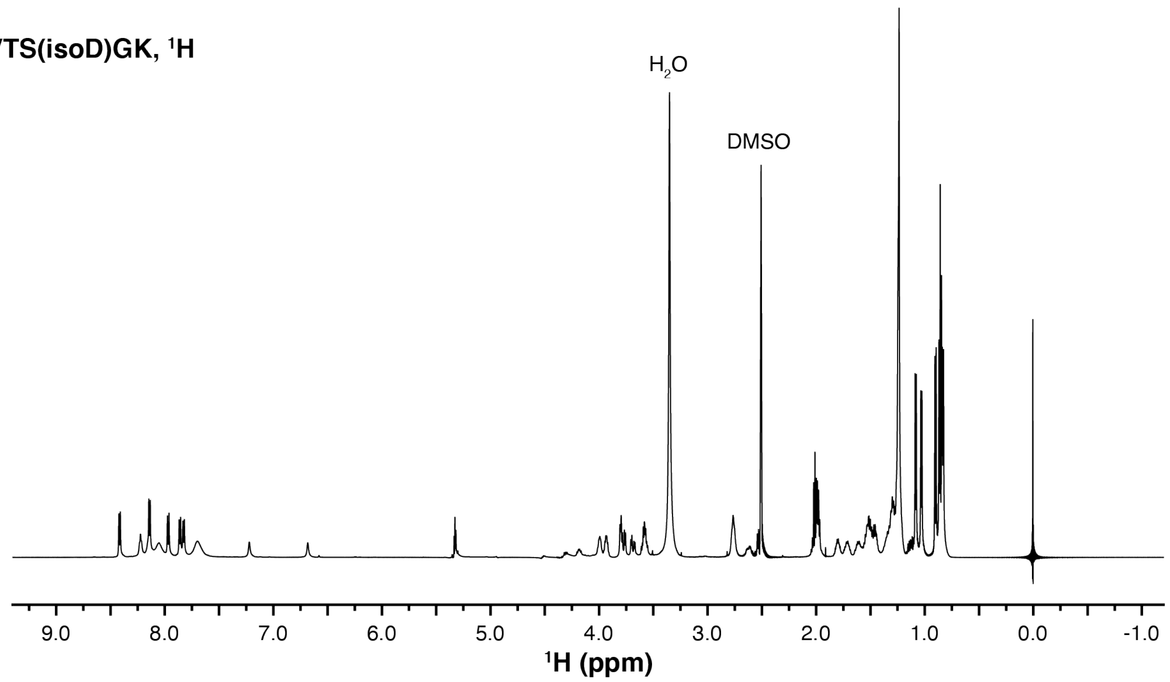

h

ITVTS(isoD)GK, ¹H-¹H COSY

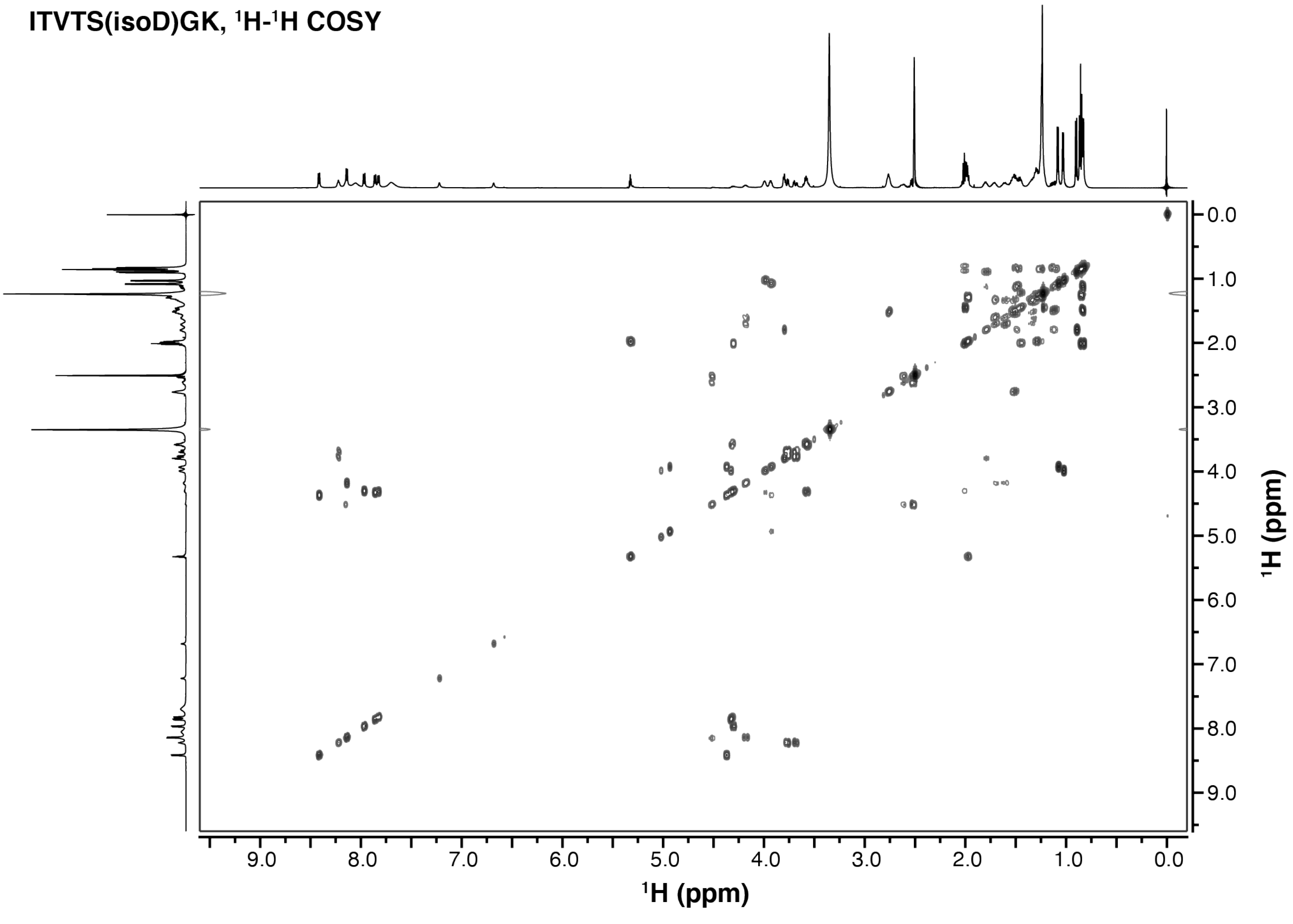

i

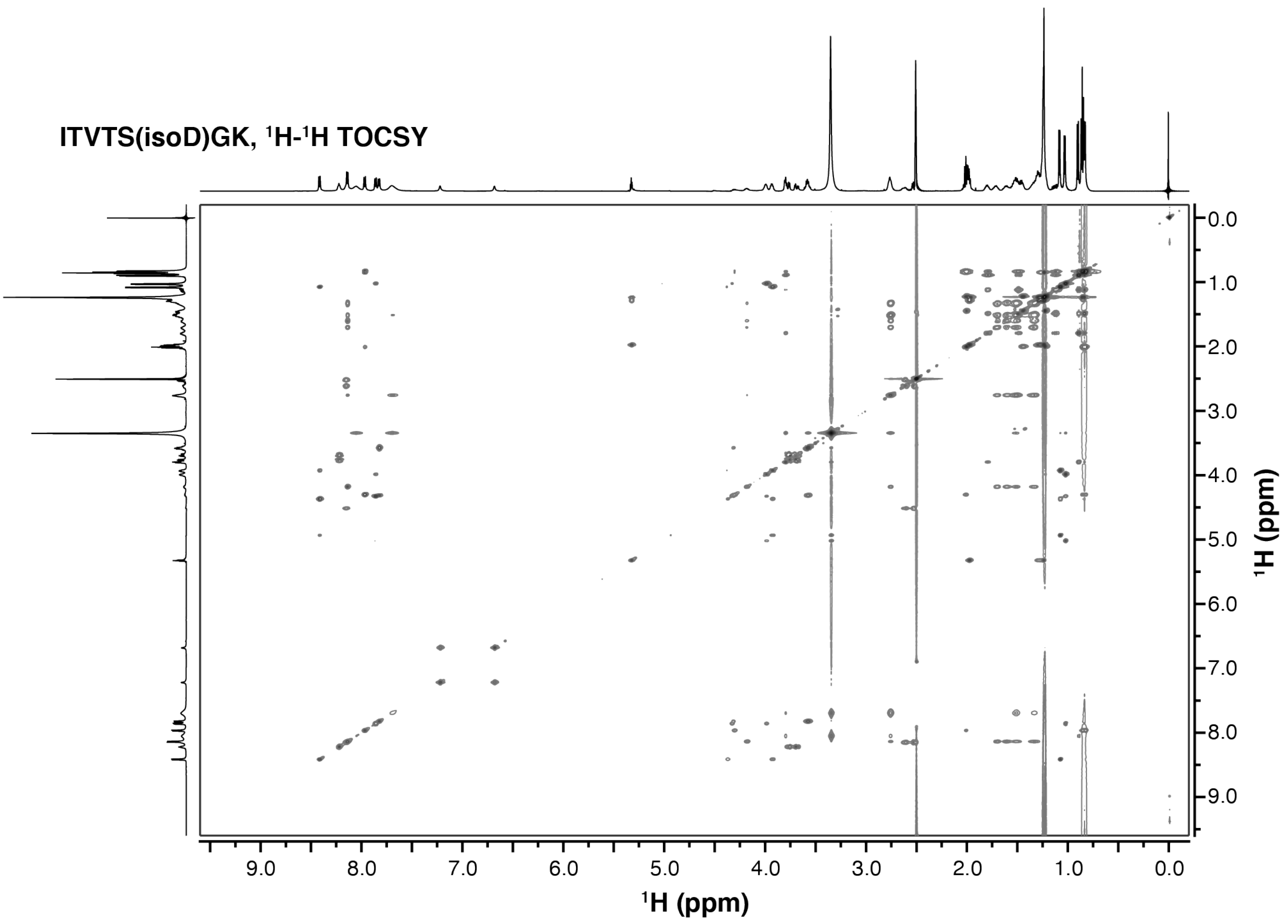
ITVTS(isoD)GK, ¹H-¹H TOCSY
0.0
1.0
2.0
3.0
4.0
5.0
6.0
7.0
8.0
9.0
¹H (ppm)
9.0 8.0 7.0 6.0 5.0 4.0 3.0 2.0 1.0 0.0
¹H (ppm)

j

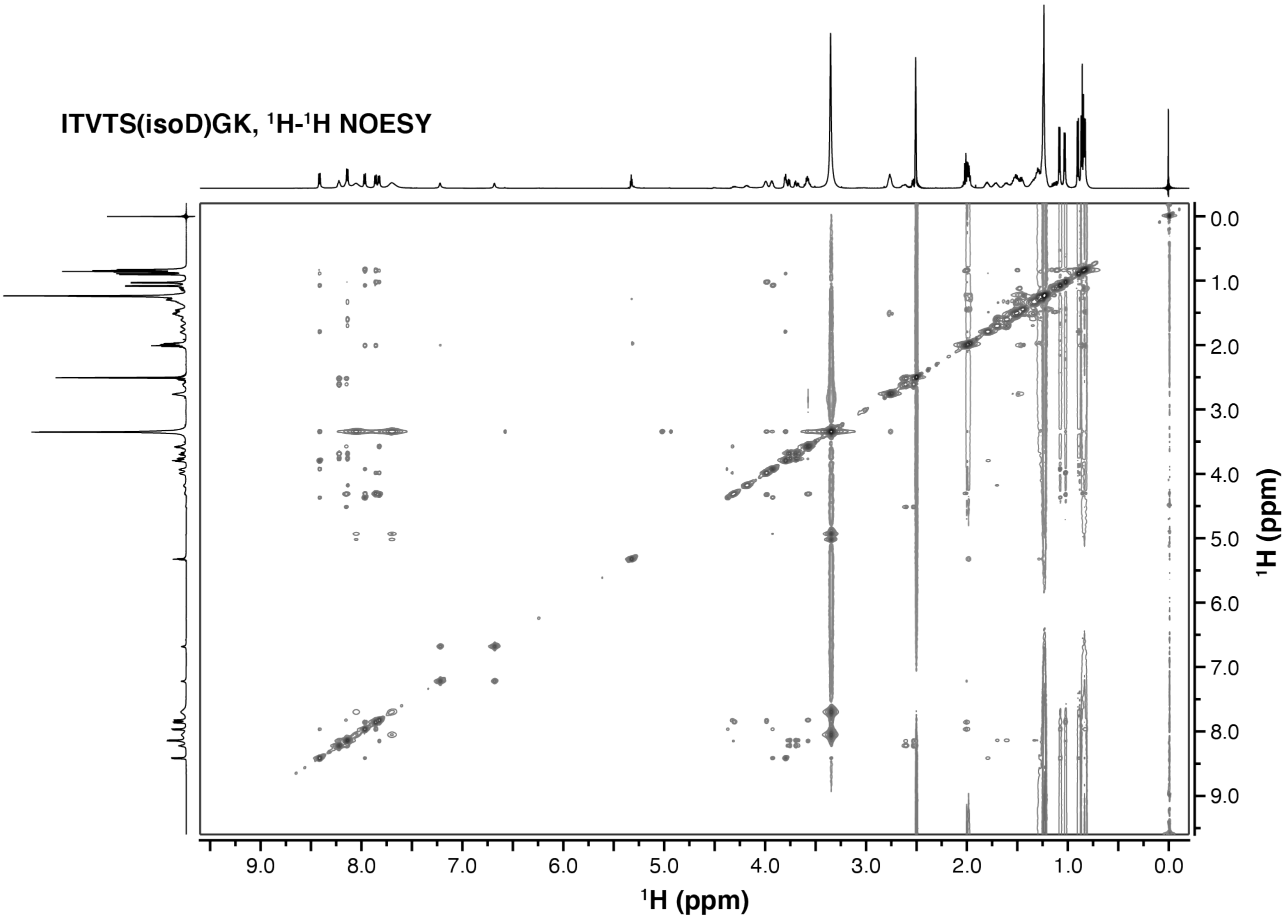
ITVTS(isoD)GK, ¹H-¹H NOESY
0.0
1.0
2.0
3.0
4.0
5.0
6.0
7.0
8.0
9.0
¹H (ppm)
9.0 8.0 7.0 6.0 5.0 4.0 3.0 2.0 1.0 0.0
¹H (ppm)

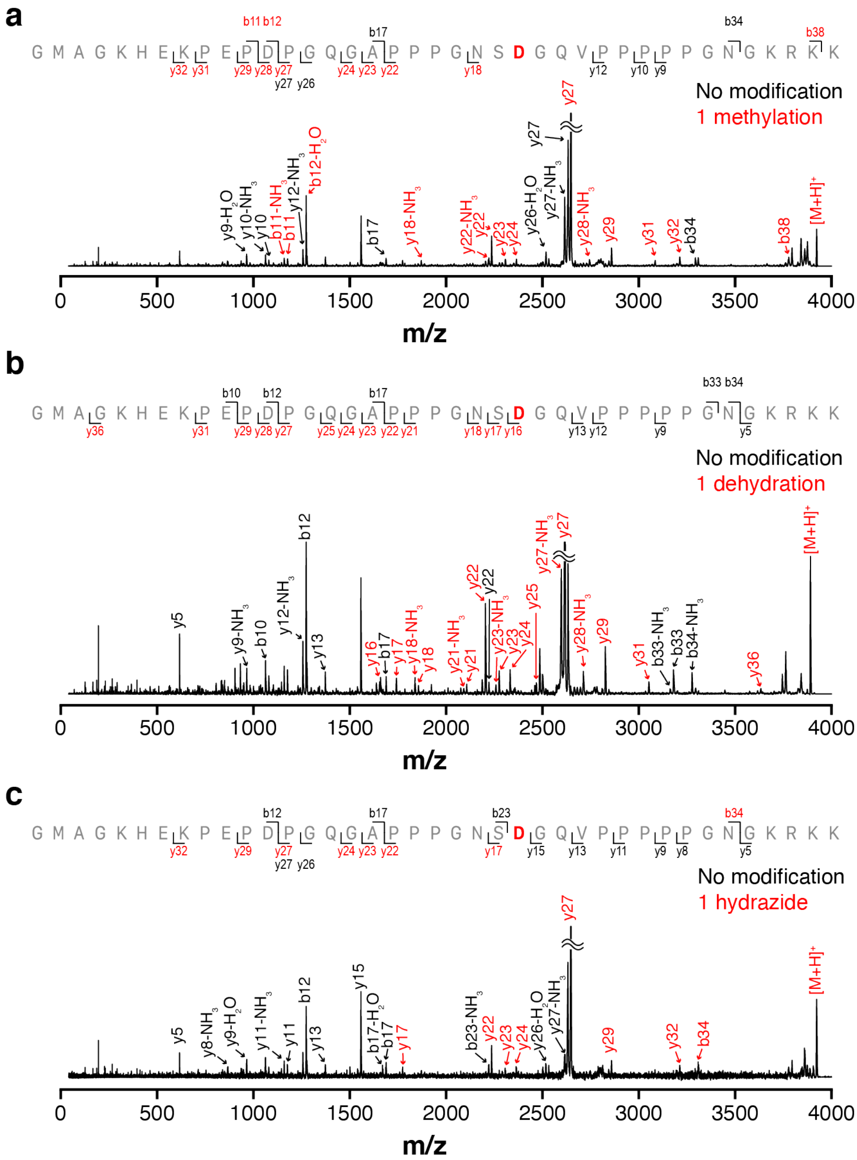

Figure S16. MS/MS analysis of SpaA derivatives. MALDI-TOF/TOF-MS spectra of methylester- (**a**), aspartimide- (**b**), or hydrazide-containing SpaA (**c**). Ions are colored according to the numbers of modified residues in the fragments (0, black; 1, red). Calculated and observed mass values can be found in Supplementary Dataset 1.

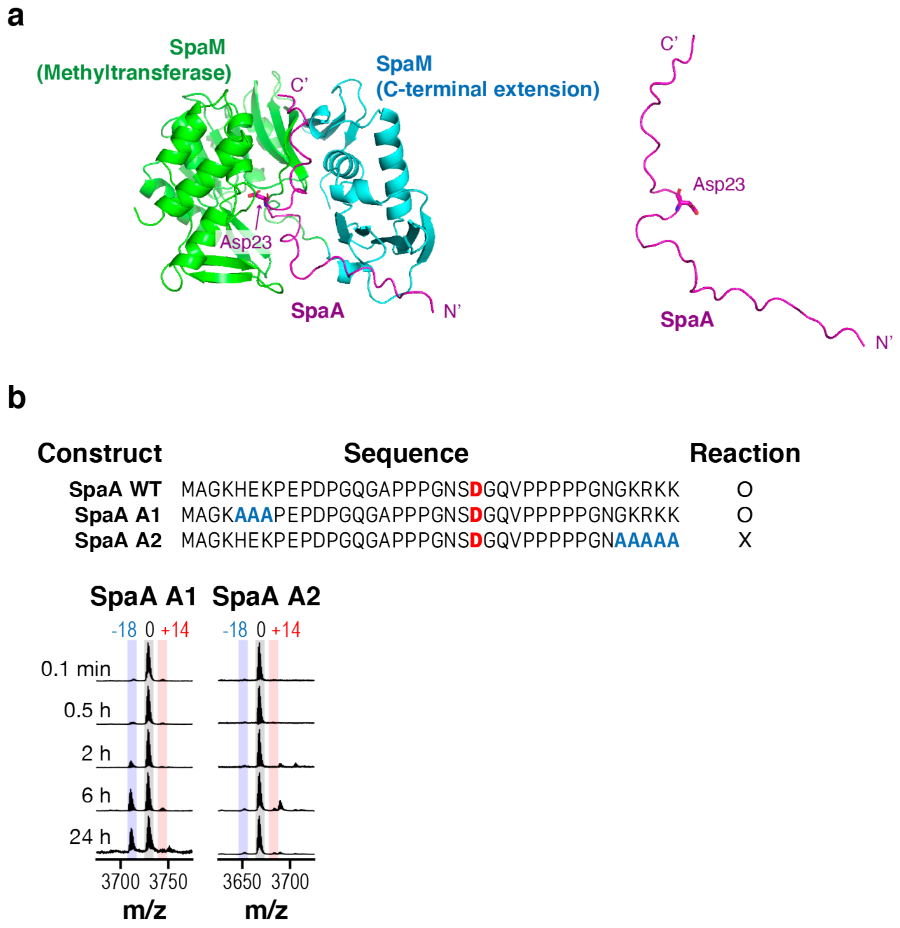

Figure S17. Model structure of SpaA and MS analysis of its variants. (**a**) AlphaFold structure of SpaA-SpaM complex (left) and standalone SpaA (right). Colors indicate the domain of the proteins (magenta, SpaA; green, SpaM methyltransferase domain; cyan, SpaM C-terminal extension). SpaM-modified aspartate (Asp23) is shown in the stick. (**b**) Sequences of alanine-substituted SpaA (SpaA A1 and SpaA A2; top) and MS spectra of the the peptides with SpaM (5 µM) and SAM (1 mM) as in Figure 5c (bottom). Relative mass values are annotated above peaks. Calculated and observed mass values can be found in Supplementary Dataset 1.

References

[1] H. Ochman, A. S. Gerber, D. L. Hartl, *Genetics* **1988**, *120*, 621-623.
[2] S. N. Ho, H. D. Hunt, R. M. Horton, J. K. Pullen, L. R. Pease, *Gene* **1989**, *77*, 51-59.
[3] H. Cho, Y. Choi, K. Min, J. B. Son, H. Park, H. H. Lee, S. Kim, *Commun. Biol.* **2020**, *3*, 547.
[4] S. Grzesiek, A. Bax, *J. Magn. Reson.* **1992**, *99*, 201-207.
[5] S. Grzesiek, A. Bax, *J. Am. Chem. Soc.* **1992**, *114*, 6291-6293.
[6] L. E. Kay, M. Ikura, R. Tschudin, A. Bax, *J. Magn. Reson.* **1990**, *89*, 496-514.
[7] R. T. Clubb, V. Thanabal, G. Wagner, *J. Magn. Reson.* **1992**, *97*, 213-217.
[8] P. Schanda, H. Van Melckebeke, B. Brutscher, *J. Am. Chem. Soc.* **2006**, *128*, 9042-9043.
[9] F. Delaglio, S. Grzesiek, G. W. Vuister, G. Zhu, J. Pfeifer, A. Bax, *J. Biomol. NMR* **1995**, *6*, 277-293.
[10] W. Lee, M. Tonelli, J. L. Markley, *Bioinformatics* **2014**, *31*, 1325-1327.
[11] L. E. Wong, J. Maier, J. Wienands, S. Becker, C. Griesinger, *J. Am. Chem. Soc.* **2018**, *140*, 3518-3522.
[12] M. R. Willcott, *J. Am. Chem. Soc.* **2009**, *131*, 13180-13180.
[13] S. F. Altschul, T. L. Madden, A. A. Schäffer, J. Zhang, Z. Zhang, W. Miller, D. J. Lipman, *Nucleic Acids Res.* **1997**, *25*, 3389-3402.
[14] K. Katoh, D. M. Standley, *Mol. Biol. Evol.* **2013**, *30*, 772-780.
[15] M. N. Price, P. S. Dehal, A. P. Arkin, *PLOS ONE* **2010**, *5*, e9490.
[16] G. A. Hudson, B. J. Burkhart, A. J. DiCaprio, C. J. Schwalen, B. Kille, T. V. Pogorelov, D. A. Mitchell, *J. Am. Chem. Soc.* **2019**, *141*, 8228-8238.
[17] J. Huerta-Cepas, F. Serra, P. Bork, *Mol. Biol. Evol.* **2016**, *33*, 1635-1638.
[18] R. Zallot, N. Oberg, J. A. Gerlt, *Biochemistry* **2019**, *58*, 4169-4182.
[19] G. E. Crooks, G. Hon, J.-M. Chandonia, S. E. Brenner, *Genome Res.* **2004**, *14*, 1188-1190.
[20] a) J. Jumper, R. Evans, A. Pritzel, T. Green, M. Figurnov, O. Ronneberger, K. Tunyasuvunakool, R. Bates, A. Žídek, A. Potapenko, A. Bridgland, C. Meyer, S. A. A. Kohl, A. J. Ballard, A. Cowie, B. Romera-Paredes, S. Nikolov, R. Jain, J. Adler, T. Back, S. Petersen, D. Reiman, E. Clancy, M. Zielinski, M. Steinegger, M. Pacholska, T. Berghammer, S. Bodenstein, D. Silver, O. Vinyals, A. W. Senior, K. Kavukcuoglu, P. Kohli, D. Hassabis, *Nature* **2021**, *596*, 583-589; b) Z. Lin, H. Akin, R. Rao, B. Hie, Z. Zhu, W. Lu, N. Smetanin, R. Verkuil, O. Kabeli, Y. Shmueli, A. dos Santos Costa, M. Fazel-Zarandi, T. Sercu, S. Candido, A. Rives, *Science* **2023**, *379*, 1123-1130.
[21] M. Mirdita, K. Schütze, Y. Moriwaki, L. Heo, S. Ovchinnikov, M. Steinegger, *Nat. Methods* **2022**, *19*, 679-682.
[22] Y.-F. Lin, C.-W. Cheng, C.-S. Shih, J.-K. Hwang, C.-S. Yu, C.-H. Lu, *J. Chem. Inf. Model* **2016**, *56*, 2287-2291.
[23] L. Holm, P. Rosenström, *Nucleic Acids Res.* **2010**, *38*, W545-W549.
[24] K. A. McCall, C. A. Fierke, *Anal. Biochem.* **2000**, *284*, 307-315.
[25] M. M. Skinner, J. M. Puvathingal, R. L. Walter, A. M. Friedman, *Structure* **2000**, *8*, 1189-1201.
[26] a) J. Z. Acedo, I. R. Bothwell, L. An, A. Trouth, C. Frazier, W. A. van der Donk, *J. Am. Chem. Soc.* **2019**, *141*, 16790-16801; b) L. Cao, M. Beiser, J. D. Koos, M. Orlova, H. E. Elashal, H. V. Schröder, A. J. Link, *J. Am. Chem. Soc.* **2021**, *143*, 11690-11702.
[27] M. Martinez-Yamout, G. B. Legge, O. Zhang, P. E. Wright, H. J. Dyson, *J. Mol. Biol.* **2000**, *300*, 805-818.

Author Contributions

S.K. supervised the overall project; H.L. and S.K. designed the study; H.L. and M.S.K. performed bioinformatic analysis; H.L., J.K., and J.L. purified peptides and proteins, and performed mass analysis and metal-binding assay; S.P. and J.H.L. designed and analyzed NMR experiments; H.L. performed structural prediction and analysis of proteins; H.L., S.P., J.H.L., and S.K. wrote the manuscript; All authors have given approval to the final version of the manuscript.
